# Rates of nonproliferative diabetic retinopathy (NPDR) regression among patients with very mild or mild NPDR in an Indian Health Service teleophthalmology program, 2015 – 2020

**DOI:** 10.1371/journal.pone.0361052

**Published:** 2026-10-16

**Authors:** Sven-Erik Bursell, Stephanie J. Fonda, Kaitlynn Borik, Jeff Stainback, Dara Shahon

**Affiliations:** 1 Estenda Solutions, Inc., Wayne, Pennsylvania, United States of America; 2 SSM Health/Saint Louis University School of Medicine, St. Louis, Missouri, United States of America; 3 Phoenix Indian Medical Center, Phoenix Arizona, United States of America; Sanmenxia Central Hospital, Henan University of Science and Technilogy, CHINA

## Abstract

Diabetic retinopathy (DR) regression is not regularly reported; yet understanding of its prevalence in early-stage DR may impact strategies for eye care management. This study’s primary aim was to examine DR regression in patients initially diagnosed with very mild or mild nonproliferative DR (NPDR). A secondary aim was to assess the association between retinopathy regression and diabetes therapy, diabetes duration, and Hemoglobin A1c (HbA1c). This was a retrospective, longitudinal cohort study using medical record data from the United States’s Indian Health Service’s teleophthalmology program for DR evaluation. Baseline exams occurred in 2015–2017 and follow-up (FU) exams occurred within 3 years of baseline. There were 2,348 patients included in this cohort with very mild NPDR at baseline and 705 patients with mild NPDR. For the whole sample, mean age was 56 ± 12.8 years, 55% were female, diabetes duration was 13.0 ± 9.1 years, and HbA1c was 8.8 ± 2.1%. Proportions for DR regression were 0.47 (CI = .45 − .50) (from very mild NPDR) and 0.36 (CI = 0.33–0.40) (from mild NPDR). Proportions for any DR progression were 0.27 (CI = 0.25–0.29) (from very mild NPDR) and 0.34 (CI = 0.30–0.38) (from mild NPDR). Multivariable analyses showed that lower HbA1c levels were associated with greater odds of diabetic retinopathy regression in patients with either very mild or mild baseline NPDR, whereas insulin use was associated with lower odds of regression. Shorter diabetes duration was associated with greater odds of regression only among patients with very mild NPDR at baseline. DR **r**egression was more frequent than DR progression particularly for patients initially with very mild NPDR. DR reversal was more likely in early-stage DR. Behavioral modifications such as increased physical activity and weight loss that lower HbA1c and subsequent risks for DR progression are important to emphasize in diabetic eye care.

## Introduction

The level of Diabetic Retinopathy (DR) is commonly used to characterize ocular microvascular complications of diabetes, the leading cause of preventable blindness amongst working-aged people [1]. Shorter durations of diabetes, improved glycemic control, and lower blood pressure and lipid levels are implicated in reducing the risk for development and progression of DR [2]. A review of prior research [3] identified studies focused on prevalence, incidence and progression of DR and the risk factors associated with them. However, regression (or improvement) of DR is less frequently studied or is a secondary focus.

Available studies on regression (Table 1) suggest it is more likely to occur in very mild nonproliferative DR (NPDR) [6,19], particularly for younger patients with hemoglobin A1c (HbA1c) levels close to normal and shorter diabetes duration [11,13,16]. Generally, the literature addressing regression focused on improvements in risk factors known to be associated with progression of DR. However, one publication [8] suggested that the formation of microaneurysms (MAs), a characteristic lesion for mild NPDR, was independent of glycemic control and other medical factors that affect more advanced levels of DR.

**Table 1:** Published studies reporting diabetic retinopathy regression.

Lead Author	Setting/Population	Study Details	Key Findings (Regression/Progression)
Caird (1962) [4]	United Kingdom single clinic population (N = 168)	Dilated retinal exam performed by an ophthalmologist with 5- years follow-up period	36% regression and 50% progression with baseline MAs only. Regression associated with younger age and better glycaemic control
Kohner (1970) [5]	United Kingdom urban eye hospitals (N = 21)	Manual MA counts from Fluorescein angiograms, 2-years follow-up	In cases with mild NPDR at baseline 22% (2 of 9) regressed to no evidence of DR. Progression not addressed.
Lee (1992) [6]	IHS facilities in Oklahoma (N = 1012)	Cohort follow up study over 12 years with 2 follow-up visits.	12.5% patients with baseline mild NPDR regressed and 25.7% progressed.
Tudor (1998) [7]	Participants in the San Luis Valley Diabetes Study, Hispanics with T2DM (N = 169)	5 years follow- up, 2-step level of DR change	17.3% regression and 24.1% progression in patients with some level of DR at baseline.
Kohner (1999) [8]	Patients enrolled in the UKPDS. (N = 2424)	MAs detected from ETDRS 7 standard photographs with follow-ups at 3, 6, 9 and 12 years	30% to 50% regression with 1–3 MAs at entry and <20% in patients presenting with 4–5 MAs. MAs > 5 related to DR progression.
Klein (2008) [9]	Wisconsin Epidemiological Study of Diabetic Retinopathy in T1DM(N = 955)	25-years follow-up and 2-step level of DR change was considered	Annual regression rate of 3.0% and progression rate of 13.5% over first 4 years of follow-up. Regression associated with higher HbA1cs and being male.
Tam (2009) [10]	Patients from a district hospital in Hongkong with T2DM (N = 413)	4-years follow-up study using a 2- step or greater change in DR level	Regression 13.2%, progression 34.7%. Mean age 55.2 years, duration 8.8 years and baseline HbA1c 8.1%. Hypertension in 60.8%. Regression associated with lower baseline A1c and no renal disease.
Varma (2009) [11]	Los Angeles Latino Eye Study (N = 775)	Wisconsin reading Center performed the retinopathy grading	Regression was 14% and progression was 38.9% in cases with DR at baseline. Fewer MAs only at baseline significantly predicted regression of DR.
Sjolie (2010) [12]	DIRECT study to study effect of candesartan on DR (N = 1905 T1DM and N = 1905 T2DM)	ETDRS level 20–47 at baseline	Higher MA score at baseline predicted increased risk for progression and reduced likelihood for regression.
Song (2011) [13]	DR screening program, School of Optometry Hong Kong (N = 5,160)	2 follow-ups over 5 years; Topcon camera used to provide 9 field 45-degree fields from each eye	45.8% regression and 6.6% progression in patients with Mild NPDR at baseline. In patients with Mod NPDR at baseline Regression was 38.9% and progression was 12.4%
Broe R (2014) [14]	Young paediatric cohort, age < 25 years at time of retinal imaging and grading 1995 (N = 339); subset re-examined in 2011 (N = 184)	Prospective study involving level of DR and other diabetes related parameters in T1 DM patients; 16-year follow-up period	Regression was 0%, progression was 64.4%, and diabetes duration at baseline was 13.5 years. Progression was associated with being male, baseline HbA1c and neuropathy.
Zavrelova (2014) [15]	T2DM patients in the Netherlands (N = 3,343)	Imaging and grading followed EURODIAB protocols, 2- to 6- years follow-up with annual visits	4.9% regressed (HbA1c of 7.8%) and 4.0% progressed (HbA1c of 8.4%). 5-year DM duration. Regression associated with lipid medication.
Jin (2014) [16]	Chinese population in Shanghai. (N = 778, 456 with DR at baseline)	5-year onset and regression of DR; imaging and grading followed EURODIAB protocols. Outcome was regression to No DR in at least 1 eye and no worsening in the fellow eye	24.2% regression to no DR over the 5-year follow-up period. Regression associated with shorter duration, lower HbA1c, normal triglycerides.
Raman (2017) [17]	Asian Indian population with DR at baseline (SN-DREAMS II). (N = 890)	Four-year FU study on incidence, progression and regression of DR	12% regression for 1-step DR change and 1.8% regression for 2 step DR change and 32% progression for 1-step DR change and 12.6% for 2-step DR change.
Lin (2022) [18]	Chinese community of people with T2DM (N = 1392)	Retinal vessel diameters were measured using IVAN and CRAE and CRVE were calculated; 2-year follow-up	10% Regression and 26.8% progression. Regression associated with treatment not involving insulin and wider CRAE.

**Notes:** MA = Microaneurysm, NPDR = nonproliferative diabetic retinopathy, T1DM = type 1 diabetes mellitus, T2DM = type 2 diabetes mellitus, DM = diabetes mellitus, CRAE = Central Retinal Artery Equivalent, IVAN = Integrative Vessel Analysis

The primary aim of this study was to investigate the occurrence of DR regression in teleophthalmology patients with initially very mild NPDR or mild NPDR. Rates of no change or stability and any progression were also determined for comparison purposes. A secondary aim was to examine the association of diabetes therapy, diabetes duration, and HbA1c with regression occurrence. Prior results on this study cohort [20,21] demonstrated prevalence of any DR, any DME, and sight-threatening disease was 28.6%, 3.0%, and 3.0%. Of patients with no DR in 2015, 18.0% had mild NPDR or worse in 2016–2019, and 0.1% had PDR. Of patients with mild NPDR in 2015, 27.2% progressed to moderate NPDR or worse in 2016–2019, and 2.3% progressed to severe NPDR or worse.

## Methods

### Setting and study population

This is a retrospective cohort study using de-identified medical records from the national, primary care-based Indian Health Service’s (IHS) teleophthalmology program (Data accessed 09/14/2022) in the United States. Baseline data included only patients with type 2 diabetes accessed to the program during the period of January 1,2015, to December 31,2015, and a follow-up period of January 1,2016, to December 31,2019. Eligible patients had at least 1 IHS teleophthalmology examination with the program in both periods.

Data from this program has previously been used to describe DR prevalence and incidence [20,21]. Briefly, the IHS teleophthalmology program is a validated American Telemedicine Association (ATA) Category 3 program [22] that accesses patients presenting for their primary care appointments [23,24]. The program’s graders are certified and licensed optometrists who use standardized protocols to identify the Early Treatment Diabetic Retinopathy Study (ETDRS) defined clinical levels of DR severity [25]. An ophthalmologist provides oversight. The study was reviewed and approved by the IHS Institutional Review Board at Phoenix Indian Medical Center (IHS IRB PXR 20.08) under the exempt process (consent waived). STROBE reporting guidelines were used for this article.

### Data selection

To establish a ‘baseline’, patients aged 20 + years who were imaged in 2015 and received a diagnosis of very mild or mild NPDR (ETDRS DR levels of severity 20 or 35) in one or both eyes were selected. Patients with no DR in both eyes, with a more severe level of NPDR than very mild or mild in one or both eyes, or with concurrent PDR (ETDRS levels 61, 65, 71−75, 81−85) and/or DME were excluded. The baseline period selected allowed time for patients to obtain a follow-up exam before possible disruptions to scheduling resulting from the COVID-19 public health emergency.

‘Follow-up’ (FU) exams were defined as the last available teleophthalmology exam in the medical record, for a minimum of 6 months and a maximum of 36 months. The average time between baseline and final FU visits was 22.8 ± 7.9 months. If the last FU exam could not be graded, the FU outcome also could not be determined (6.9% of patients with a FU exam had to be excluded from calculations of the outcomes).

### Measures

**Assessment of DR severity**. Over 80% of the DR examinations used nonmydriatic ultra-widefield imaging (UWFI) scanning laser ophthalmoscopy (Daytona, Optos, plc, Dunfermline, United Kingdom). The UWFI protocol included non-simultaneous stereo pair 200^o^ images from each eye, centered on the macula. The remaining examinations used low-illumination, nonmydriatic fundus photography (NMFP) digital imaging system (Topcon NW6S; Topcon Medical Systems, Inc., Paramus, NJ) with a custom digital camera back (Megavision Retinal Image Capture; Santa Barbara, CA). The NMFP protocol included three non-simultaneous stereo pair 45^o^ (degree) images from different retinal regions and two non-simultaneous stereo pair 30^o^ digital images of the optic disc and the macula from the retina of each eye were obtained, for an approximate total retinal coverage of 90^o^ to 135^o^. These methods and their agreement with ETDRS photography have been previously described [26,27].

**DR outcomes.** For the patients with very mild NPDR at baseline, the outcome had four categories based on the patients’ worst eye: regression to no DR; no change in DR; progression to mild NPDR and progression to any higher level of DR as of the patients’ last FU exam. Progression to mild NPDR was considered separately to assess whether these patients differed from those whose diagnosis at FU was still very mild NPDR. For patients with mild NPDR at baseline, the outcome had four categories based on the patients’ worst eye: regression to no DR; regression to very mild NPDR; no change in DR; and progression to any higher level of DR. For some statistical analyses, no change and any DR progression were combined into one group grouped for comparison with any regression.

**Background measures.** Background measures [28] included age (years), sex, diabetes therapy; diet only such as Mediterranean diet, reducing refined carbohydrates/sugars, increasing fiber, and more plant-based eating; oral diabetes medications but not insulin; any insulin with or without oral diabetes medications; or unknown/unspecified in the record, self-reported diabetes duration (years), and baseline HbA1c (%). If patients were missing a value for baseline HbA1c, then the FU HbA1c value was substituted, and if HbA1c data were unavailable at both baseline and FU, then the mean value of the cohort was substituted.

### Statistical analysis

The analysis documents the totals and means or percentages for the patients’ background characteristics. Additionally, to understand possible biases in the cohort with FU data, all the selected background characteristics were compared between patients with a FU exam versus those who did not have one. Continuous variables (age, diabetes duration, HbA1c) were analyzed using independent samples t-tests, and categorical variables (sex, diabetes therapy) were analyzed using Chi-square tests. The very mild and mild NPDR baseline cohorts were combined for this analysis.

For the first study aim, the analysis obtained the numbers and proportions [with 95% multinomial confidence intervals (CIs)] of patients with each DR outcome. Chi-square tests for equal proportions were applied. For the second study aim unadjusted proportions or means (with CIs) for diabetes therapy groups, diabetes duration, and HbA1c were also obtained by DR outcome. Then, to account for potential confounding among these background factors, multivariable logistic regression models were used to evaluate their independent associations with DR regression; DR regression versus all other DR outcomes was a binary outcome and age, sex, diabetes therapy, diabetes duration and HbA1c were covariates. Using the multivariable logistic regression results, we then calculated predicted probabilities at specific HbA1c levels and whether patients were on oral medication only or insulin, holding age constant at 60 years, diabetes duration at 10 years and sex as female. The very mild and mild NPDR patients were separated for all these analyses.

Statistical significance was set at p < .01 to reduce Type 1 error and identify more meaningful associations. The analyses used SAS software Version 9.4 for Windows, Copyright © 2020 (SAS Institute Inc., Cary, NC, USA).

## Results

There were 5,735 patients who met the initial selection criteria; 4,367 (76.1%) had very mild NPDR and 1,368 (23.8%) had mild NPDR. Of these, 2,348 (53.7%) with very mild NPDR and 705 (51.5%) with mild NPDR had a FU exam within 6–36 months post baseline.

On average, the patients who had a FU exam were 56.0 ± 12.8- years of age and had had diabetes for 13.0 ± 9.1 years at the time of their baseline exam (Table 2). Forty one percent of these patients were on oral diabetes medications (only) and 45% were male. Their average HbA1c was 8.8 ± 2.2%.

**Table 2:** Baseline characteristics of patients with very Mild/Mild NPDR at baseline, overall and by whether they had a Follow-up ExamExam.

Baseline Characteristic	All, Irrespective of FU	Had FU Exam	No FU Exam	p value
Patients, No. (%)	5735 (100)	3053 (53.2)	2682 (46.8)	NA
				
Very mild or mild NPDR, No. (col %)				
Very mild	4367 (76.1)	2348 (76.9)	2019 (75.3)	.15
Mild	1368 (23.8)	705 (23.1)	663 (24.7)	
				
Years of age, mean (SD)	56.4 (13.3)	56.0 (12.8)	56.8 (13.9)	.03
				
Sex, No. (col %)				
Male	2571 (44.8)	1373 (45.0)	1198 (44.7)	.82
Female	3164 (55.2)	1680 (55.0)	1484 (55.3)	
				
Diabetes therapy, No. (col %)				
Diet only	222 (3.6)	107 (3.5)	115 (4.3)	.03
Oral medication (only)	2306 (38.5)	1265 (41.4)	1041 (38.8)	
Insulin	2661 (49.4)	1415 (46.4)	1246 (46.5)	
Unspecified	546 (8.5)	266 (8.7)	280 (10.4)	
				
Diabetes duration years, mean (SD)	13.3 (9.3)	13.0 (9.1)	13.8 (9.6)	.003
				
Baseline HbA1c %, mean (SD)	8.8 (2.1)	8.8 (2.1)	8.8 (2.1)	.50

**Notes**: P values are for t or chi-square tests comparing characteristics of patients with or without FU exams. Table includes patients who had an ungradable exam at FU. Patients on insulin could have been taking it with oral medications or alone. NPDR = nonproliferative diabetic retinopathy, FU = follow-up, SD = standard deviation, col = column, NA = not applicable, and No. = number.

In the group with very mild NPDR at baseline, the proportions for regression to no DR, no change, progression to mild NPDR and progression to levels of DR more advanced than mild NPDR were 0.47 (95% CI = 0.45–0.50), 0.26 (95% CI = 0.23–0 .28), .08 (95% CI .06 −.10) and 0.19 (95% CI = 0.17–0.22), respectively (Table 3). In the group with mild NPDR at baseline, the proportions for regression to no DR, to very mild DR, no change and progression were 0.16 (95% CI = 0.12–0.20), .20 (95% CI = .16 − .24), .30 (95% CI = 0.26–0.34) and 0.34 (95% CI = 0.30–0.38) (Table 4). Chi-square tests for equal proportions, indicated that the proportions for the DR outcomes differed statistically for both patient groups (p < .0001). However, in the group with mild NPDR at baseline, the CIs for regression to no DR and regression to very mild NPDR overlapped, suggesting these proportions were similar. Thus, combining the two levels of regression for mild NPDR patients, the proportion with any regression was .36 (95% CI = 0.33–0.40).

**Table 3:** Unadjusted outcomes and outcomes by selected background characteristics in patients with very mild NPDR at baseline.

	Follow-up Outcomes			
	Regression to No DR	No change	Progression to Mild NPDR	Progression to> Mild NPDR
**Unadjusted**				
No. (proportion; 95% CI) ^1^	1042 (.47; .45 − .50)	562 (.26; .23 − .28)	170 (.08; .06 − .10)	427 (.19; .17 − .22)
				
**Background Characteristic**				
Diabetes therapy, No. (row proportion; 95% CI)				
Diet only	61 (.71; .63 − .81)	16 (.19; .10 − .29)	3 (.03; .00 − .14)	6 (.07; .00 − .17)
Oral medication (only)	549 (.56; .53 − .60)	224 (.23; .20 − .26)	59 (.06; .03 − .09)	143 (.15; .11 − .18)
Insulin (with or without oral meds)	337 (.35; .32 − .39)	272 (.10; .07 − .14)	98 (.10; .07 − .14)	247 (.26; .23 − .29)
Unspecified	95 (.51; .44 − .59)	50 (.27; .20 − .35)	10 (.05; .00 − .13)	31 (.16; .10 − .25)
				
Diabetes duration years, mean (SD; 95% CI)	9.9 (8.1; 9.4 - 10.4)	13.2 (8.6; 12.4 - 14.0)	15.7 (9.1; 14.2 - 17.1)	13.1 (8.2; 12.3 - 14.0)
				
HbA1c %, mean (SD; 95% CI)	8.3 (2.0; 8.2 - 8.5)	8.7 (1.9; 8.5 - 8.8)	9.1 (2.2; 8.8 - 9.5)	9.9 (2.0; 9.7 - 10.1)

**Notes:** ^1^ Chi-square tests for equal proportions, indicated that the proportions for the DR outcomes differed statistically (p < .0001).

Table excludes patients with ungradable images at follow-up. Patients on insulin could have been taking it with oral medications or alone. NPDR = nonproliferative diabetic retinopathy, No. = number, CI = confidence intervals, and SD = standard deviation.

**Table 4:** Unadjusted outcomes and outcomes by selected background characteristics in patients with mild NPDR at baseline.

	Follow-up Outcomes			
	Regression to No DR	Regression to Very Mild NPDR	No change	Any Progression
**Unadjusted**				
No. (proportion; 95% CI) ^1^	104 (.16; .12 − .20)	128 (.20; .16 − .24)	190 (.30; .26 − .34)	219 (.34; .30 − .38)
				
**Background Characteristic**				
Diabetes therapy, No. (row prop; 95% CI)				
Diet only	4 (.25; .06 − .55)	2 (.13; .00 − .41)	4 (.25; .06 − .54)	6 (.38; .19 − .67)
Oral medication (only)	50 (.23; .16 − .30)	51 (.23; .16 − .30)	61 (.28; .21 − .35)	57 (.26; .19 − .33)
Insulin (with or without oral meds)	39 (.11; .06 − .17)	65 (.19; .13 − .24)	111 (.32; .26 − .37)	136 (.39; .33 − .44)
Unspecified	11 (.20; .07 − .35)	10 (.18; .05 − .32)	14 (.25; .13 − .40)	20 (.36; .24 − .51)
				
Diabetes duration years, mean (SD; 95% CI)	12.4 (9.8; 10.4 - 14.4)	16.0 (10.6; 14.1 - 17.9)	16.7 (10.1; 15.2 - 18.2)	15.9 (8.4; 14.7 - 17.0)
				
HbA1c %, mean (SD; 95% CI)	8.3 (2.0; 7.9 - 8.7)	8.5 (2.0; 8.2 - 8.9)	8.6 (1.8; 8.3 - 8.9)	9.6 (2.0; 9.3 - 9.8)

**Notes:** ^1^ Chi-square tests for equal proportions, indicated that the proportions for the DR outcomes differed statistically (p < .0001).

Table excludes patients with ungradable images at follow-up. Patients on insulin could have been taking it with oral medications or alone. NPDR = nonproliferative diabetic retinopathy, No. = number, CI = confidence intervals, prop = proportion, and SD = standard deviation

For patients with very mild NPDR, the proportion of patients on ‘diet only’ or oral medications at baseline, who regressed, was 0.71 (95% CI = 0.63–0.81) and 0.56 (95%vCI = 0.53–0.60) whereas the proportion of patients taking insulin who regressed was 0.35; 95% CI = 0.32–0.39. Mean diabetes duration was 9.9 ± 8.1 years (95% CI = 9.4–10.4) for regressors, lower than for patients with all other DR outcomes. Mean HbA1c was 8.3 ± 2.0% (95% CI = 8.2–8.5) for regressors versus 8.7 ± 1.9% (95% CI = 8.5–8.8) for patients with no change, 9.1 ± 2.2% (95% CI = 8.8–9.5) for patients progressing to mild NPDR and 9.9 ± 2.0% (95% CI = 9.7–10.1) for progression to more advanced levels of DR.

Patients with mild NPDR at baseline and treated with insulin had lower proportions of any regression (0.30; 95% CI = 0.24–0.35) compared with those treated with oral agents (proportion for any regression (0.46; 95% CI = 0.39–0.53) (Table 4). Patients who experienced regression had shorter diabetes duration on average (12.4 ± 9.8 years; 95% CI = 10.4–14.4), whereas those with no change or progression had longer duration, on average (approximately 16 years or more). Higher HbA1c levels were associated with worse outcomes, with the highest mean HbA1c observed among patients with progression; however, HbA1c levels were similar for patients whose DR regressed or did not change.

The multivariable logistic regression model with DR regression (vs all other DR outcomes) as the outcome, showed that the odds of regression were slightly higher with increasing age (odds ratio [OR] per year = 1.02; 95% CI, 1.02–1.02) for patients with very mild NPDR at baseline (Table 5). In contrast, the odds of regression were lower among patients treated with insulin compared with oral agents (OR = 0.61; 95% CI, 0.50–0.75), with longer diabetes duration (OR per year = 0.95; 95% CI, 0.94–0.96), and with higher HbA1c (OR per percentage point = 0.86; 95% CI, 0.82–0.90).

**Table 5:** Adjusted odds ratios for diabetic retinopathy regression (vs All Other Outcomes) by baseline NPDR severity.

	Very Mild NPDR at Baseline				Mild NPDR at Baseline			
Parameter	Odds	95% Confidence Interval		p value	Odds	95% Confidence Interval		p value
Intercept	NA	NA	NA	.0003	NA	NA	NA	.54
Male	0.85	0.70	1.03	.09	0.77	0.54	1.10	.14
Years of age	1.02	1.01	1.02	.0002	1.02	1.00	1.03	.02
Diabetes therapy								
Diet only	1.71	0.97	3.01	.06	0.67	0.22	2.07	.48
Oral medication (only)	Ref	Ref	Ref	Ref	Ref	Ref	Ref	Ref
Insulin (with or without oral meds)	0.61	0.50	0.75	<.0001	0.54	0.37	0.79	.002
Unspecified	0.98	0.66	1.46	.92	0.99	0.49	2.01	.98
Diabetes duration years	0.95	0.94	0.96	<.0001	0.98	0.96	1.00	.09
HbA1c %	0.86	0.82	0.90	<.0001	0.87	0.79	0.95	.003

Notes

Among patients with very mild NPDR at baseline, the model demonstrated moderate discrimination (c-statistic [AUC] = 0.69) and adequate calibration (Hosmer–Lemeshow p = 0.22). Model fit statistics were HbAIc = 2548.8 and −2 log likelihood = 2532.8.Among patients with mild NPDR at baseline, model performance was lower (c-statistic [AUC] = 0.65), although calibration remained adequate (Hosmer–Lemeshow p = 0.32); HbAIc = 750.2 and −2 log likelihood = 734.2.NA = not applicable; Ref = reference category.

Among patients with mild NPDR at baseline, the pattern was similar: the odds of regression were reduced with insulin use (OR = 0.54; 95% CI, 0.37–0.79) and higher HbA1c (OR per percentage point = 0.87; 95% CI, 0.79–0.95).

The model-based predicted probabilities (Table 6) illustrate how the probability of DR regression decreased progressively with increasing HbA1c and was consistently lower among patients treated with insulin than patients treated with oral agents only. At any given HbA1c level, predicted probabilities of regression were also lower among patients with mild NPDR at baseline than among those with very mild NPDR. For example, at an HbA1c of 8%, the predicted probability of regression among patients with very mild NPDR was 61% for those treated with oral agents versus 49% for those using insulin; corresponding probabilities among patients with mild NPDR were 53% and 38%, respectively.

**Table 6:** Adjusted predicted probabilities of diabetic retinopathy regression at selected HbA1c values, by baseline NPDR severity.

	Very Mild NPDR at Baseline		Mild NPDR at Baseline	
HbA1c %	Oral Medication Only	Any Insulin	Oral Medication Only	Any Insulin
6	0.68	0.57	0.60	0.45
6.5	0.67	0.55	0.59	0.43
7	0.65	0.53	0.57	0.42
7.5	0.63	0.51	0.55	0.40
8	0.61	0.49	0.53	0.38
8.5	0.60	0.47	0.52	0.36
9	0.58	0.45	0.50	0.35
9.5	0.56	0.43	0.48	0.33
10	0.54	0.42	0.46	0.32
10.5	0.52	0.40	0.44	0.30
11	0.50	0.38	0.43	0.28
11.5	0.48	0.36	0.41	0.27
12	0.46	0.34	0.39	0.26

Notes

Predicted probabilities were estimated from logistic regression models including age (years), sex, diabetes duration (years), diabetes therapy category, and HbA1c (%) as covariates. The outcome was regression versus all other diabetic retinopathy outcomes. Values represent predicted probabilities at selected A1c levels, with other covariates held constant at representative values (age = 60 years, diabetes duration = 10 years, male = 0, female = 1).

## Discussion

This study looked at DR outcomes over time for patients initially diagnosed with very mild or mild NPDR. Our focus was how often regression occurred for these patients, but we also looked at no change/stability and any progression for comparison. Patients with very mild baseline NPDR had substantially more regression to no DR (proportion = 0.47; 95% CI = 0.45–0.50) than no change/stability (proportion = 0.33; 95% CI = 0.31–0.36). Further, the proportion for regression was more than twice that for progression (0.19; 95% CI = 0.17–0.22). For patients with mild baseline NPDR, the proportion that had any regression (to no DR or to very mild NPDR) was 0.36 (95% CI = 0.33–0.40), which was higher than the other DR outcomes for this group yet lower than that observed for the very mild NPDR group. The proportion of patients with baseline mild NPDR who regressed to no DR was 0.16 (95% CI = 0.12–0.20). Thus, the likelihood of improvement diminishes substantially from mild NPDR.

For the very mild NPDR group, regression of DR was predictably reduced for patients with higher HbA1c, longer diabetes duration, and insulin use. Similarly, for the mild NPDR group, regression of DR was reduced for patients with higher HbA1c levels and insulin use. Predicted probabilities obtained from multivariable logistic regression models highlight how HbA1c and insulin use have a synergistic effect on the chances of DR regression. We believe that insulin use per se is not the cause of DR progression. Rather, baseline insulin use may be an indication that glycemic control was historically poor and/or insulin was initiated after DR improvement was less possible. This hypothesis cannot be tested with these data, however.

Most publications have reported higher rates of progression than regression with baseline mild NPDR (Table 1) [4,6,7,9–11,13–16,18]. When MA counts were used as the DR metric (9,27,28), regression rates of between 20% to 50% were noted for very mild NPDR (< 5 MAs and no IRMA or venous beading). For MA counts > 5 (mild NPDR) there was an increased likelihood for progression comparable to the results from this study. Generally, these published studies included older patients with mean age > 50 years and longer durations of diabetes. In contrast, a study on a cohort with mean age < 25 years [14] found 0% regression and 64.4% progression suggesting that younger aged patients were more likely to progress in DR than regress. A Singapore-based optometry practice [13] reported a regression rate of 45.5% and a progression rate of 6.6% in older patients (mean age > 63 years) with mild baseline NPDR. This article highlights the probability of higher rates of regression than progression from mild NPDR, consistent with the results from our study. However, it was notable that the distinction between very mild and mild NPDR was not addressed, and no data was available on baseline clinical metrics such as HbA1c or duration of diabetes.

Overall, the mean HbA1c in these IHS patients was greater than 8%, higher than the American Diabetes Association recommended target of 7% [29]. The literature on progression would suggest that this cohort would have more progression than regression. Yet the rates of regression we found suggest that improving glycemic control may not be the primary cause of regression in this cohort. In a different cohort, Kohner [8] found that the formation rate of MAs is independent of blood glucose control and other medical variables suggesting that other factors, beyond improving the risk factors for progression, may play a role in the observed regression rates. Further research into other factors potentially affecting DR regression is needed. Additional factors could include behavioral components such as improved nutrition or increased physical activity and treatments to reduce oxidative stress and inflammatory cytokine levels. Medications such as Candesartan [30] and Fenofibrate [31] that have been shown to be effective in reducing progression of DR also have a positive effect on promoting DR regression from levels of mild and moderate NPDR, independent of factors such as HbA1c. There are also naturally occurring proteins in the retina and vitreous such as Retinal Binding Protein-3 (RBP-3) [32] and Lysophosphatidic acid (LPA) [33] where higher levels are associated with less severe DR and resistance to DR progression. Higher retinal RBP-3 and vitreous LPA concentrations could explain the observed intrinsic regression activity. The vascular architecture may also have characteristics that favor regression [18] where a measure characterizing retinal artery diameter, was significantly larger (less flow resistance, and less mechanical stress on the retinal vascular endothelial cells) and associated with DR regression.

A strength of this primary care-based, teleophthalmology study is the wide US geodiversity where 53% of the patients returned for at least one FU visit within 36 months. A centralized reading center, standardized protocols to diagnose DR severity, and strict quality control measures used by the teleophthalmology program minimized measurement error. This is also the first large study to differentiate DR change in patients with very mild NPDR from those with mild NPDR, which can further our understanding of regression even though treatment and exam protocols do not differ for these levels of DR.

A possible criticism of this study is that the proportions for the outcomes observed were due to inconsistencies in the severity grading of DR over time rather than to “real change”. Of note for this study, is that the level of DR assigned to the patient is the DR level of the worst eye. So, for a regression outcome there needs to be no evidence of DR in both eyes and MA turnover would have minimal impact on the DR study outcome level. To address the potential grading inconsistency, we performed an identical analysis on those patients who had the same diagnoses for two consecutive FU exams [N = 666/3053 (21.9%)]. We found that the proportions for the DR outcomes and their associations with the background clinical metrics were very similar. For example, in the very mild NPDR group, the proportions for regression to no DR, no change, and progression were 0.45 (95% CI = 0.41–0 .50) 0.40 (95% CI = 0.36–0.45), and 0.15 (95% CI = 0.10–0.19), respectively. Agreement between the subset analysis and what we have reported for the whole group held for the mild NPDR group as well. The concordance provided confidence that any inconsistencies in DR grading over time would minimally impact the study results.

Another limitation of the study is that 7 percent of the patients who had a FU exam had retinal images that could not be graded (6% of patients with very mild NPDR and 9% of patients with mild NPDR at baseline), which may have affected the estimate of regression. These patients were slightly older (average age was 62.5 years for patients with mild NPDR at baseline) and proportionally more of them were taking insulin (50–51% versus 46.4% of patients who had gradable retinal images). Average HbA1c for these patients was the same as that of patients with gradable images. Thus, it is possible that not having diagnoses for these patients might have led to a minor underestimation of progression, though not enough to impact the finding that regression was more common than progression among these patients.

The main limitation to this study was that there was no access to medical record information such as BMI, clinical laboratory values beyond A1c, or prescribed medications for the whole cohort, as the teleophthalmology program is still undergoing integration with the patients’ main electronic medical records. Also, approximately 47% of patients did not have a FU teleophthalmology exam within 6–36 months of their baseline exam, potentially affecting the estimate of regression occurrence. However, comparison of the background characteristics of the followed and not followed groups found they were statistically similar except for mean diabetes duration, which was slightly shorter for the followed group (Table 2; 13.0 vs. 13.8 years, p = .003).

In summary, we have shown that regression is most likely to occur in cases with very mild NPDR at baseline although regression from mild NPDR at baseline is also notable and is consistent with our recent prior studies showing lower DR incidence and prevalence rates [20,21] compared to earlier IHS-based studies. A contributing factor impacting the high regression rates and the slower rates of progression may be associated with the success of the IHS Special Diabetes Program for Indians (SDPI) [34]. A 2022 SDPI report (302 sites across 35 US states), demonstrates that the program delivers culturally sensitive health services, including counselling in nutrition, physical activity and weight management, to over 80% of their patients. For most Americans the American Diabetes Association recommends an A1c goal of <7% [29]. However, the IHS recognizes the importance of individualizing A1c goals when appropriate and has set a clinical target goal of < 8%. The IHS also recognizes that tighter goals for younger and healthier people (SDPI youth services) may be appropriate, and less stringent goals may be considered for older patients [34]. The IHS clinical guideline for A1c is supported by the results from this study. The study data suggest optimizing health service delivery through allocation of more healthcare resources to the management of younger, potentially higher risk patients in primary care. Additionally, although more research is needed to understand what/how factors contribute to regression per se, its high rate can be presented as a positive motivational message [35] for promoting healthy behavior changes to patients seeking to manage their diabetes and subsequent eye health through behavior and lifestyle, especially older patients [36].
