# Supplementary material for: Rates of nonproliferative diabetic retinopathy (NPDR) regression among patients with very mild or mild NPDR in an Indian Health Service teleophthalmology program, 2015 – 2020
Source: PLoS One. 2026 Oct 16;21(10):e0361052. doi: 10.1371/journal.pone.0361052 (PMC13674746; doi:10.1371/journal.pone.0361052)
Supplement: Supplementary file 1 [file pone.0361052.s002.docx]

May 19, 2026

Dear PLOS Editor(s) and Reviewers,

My co-authors and I are submitting our revised manuscript, “**Rates of Nonproliferative Diabetic Retinopathy (NPDR) regression among patients with Very Mild or Mild NPDR in an Indian Health Service teleophthalmology program, 2015 - 2020**” for further consideration and publication in *PLOS*.

We would like to thank the reviewers for their insightful comments. This process has substantially strengthened manuscript.

This document lists each comment provided by the editorial staff and reviewers with our responses immediately below, italicized. Please do not hesitate to request clarifications.

**Summary of changes:**

In response to comments from Reviewer 1 regarding the need for a multivariable logistic regression analysis, we performed the suggested analysis presented the results in a new table (Table 5). To ground these results, we additionally calculated model-based predicted probabilities in presented these in another new table (Table 6). This latter table illustrates how the probability of DR regression decreased progressively with increasing HbA1c, by diabetes treatment categories. Due to the multivariable logistic regressions, the p-value columns in Table 3 and Table 4 from previous bivariate analyses became redundant and have been deleted.

In the original version of Table 3, we combined the outcome of “any progression from very mild NPDR” as a single group. Given reviewer comments, it seemed important to differentiate between “progression from very mild NPDR to mild NPDR” and “progression from very mild NPDR to DR levels greater than mild NPDR”. This differentiation is now shown in the revised Table 3.

.

The underlying data base has been published in the Harvard Dataverse repository and is publicly available at the following link: <https://doi.org/10.7910/DVN/Q5HFML>

**Upload to protocols.io if applicable:**

*This is not applicable to this manuscript*

**ORCID ID Numbers**

*Completed for all authors who have ORCID ID numbers*

**Journal Requirements:**

1. When submitting your revision, we need you to address these additional requirements.

*PLOS ONE's style/format requirements have been implemented as outlined in the PLOS ONE style templates.*

2. Thank you for stating the following in the Acknowledgments Section of your manuscript:

This material is the result of work supported with resources and the use of facilities at the Indian Health Service Teleophthalmology Program, Joslin Vision Network, located in the Phoenix Area of the Indian Health Service. The contents of this article do not represent the views of the Indian Health Service or the United States Government.

*The Acknowledgement Section is amended to read as follows “The content of this article does not represent the views of the Indian Health Service or the United States Government.”*

*Amended Funding Statement as follows: “The funder, Estenda Solutions, a commercial company, provided support in the form of author salaries and research materials but did not have any additional role in the study design, data collection and analysis, decision to publish, or preparation of the manuscript. The specific roles of these authors are articulated in the ‘author contributions’ section.”*

3. Competing Interests section.

*Author Bursell declared SocialEyes Corp as a potential competing interest. As this consulting position is unpaid with no other financial associations it does not need to be declared here so it has been removed from the Competing Interests section.*

***Updated Competing Interests statement:***

*I have read the journal's policy and the authors of this manuscript have the following competing interests:*

*Sven-Erik Bursell: Estenda Solutions Research Consultant subcontracted by the Indian Health Service Joslin Vision Network to manage and analyze the data collected for the program.*

*Stephanie J. Fonda: Subcontracted by the Indian Health Service Joslin Vision Network to manage and analyze the data collected for the program as Estenda Solutions Vice President of Research Initiatives.*

*Jeff Stainback: Subcontracted by the Indian Health Service Joslin Vision Network to manage and analyze the data collected for the program as Estenda Solutions Quality Assurance Manager.*

*Dara Shahon: Employee of the Indian Health Service as Director, IHS-JVN Teleophthalmology Program at the Phoenix Indian Medical Center.*

*This does not alter our adherence to PLOS ONE policies on sharing data and materials.*

5. We note that you have indicated that there are restrictions to data sharing for this study.

*These restrictions have been resolved and through the IHS National IRB committee and the Phoenix Area IHS IRB committee we have received approval to publicly share the anonymized underlying data associated with this study.*

*Thus, there are no longer any restrictions on data sharing* *and our Data Availability statement in the submission form will be updated accordingly.*

7. Reviewer comments include a recommendation to cite specific previously published work.

*Reviewer 2 recommended citation of a DR study from Bhutan (*[*https://doi.org/10.1007/s10384-023-00989-6*](https://doi.org/10.1007/s10384-023-00989-6)*) that documented DR prevalence and severity but not regression. Based on Reviewer 2 comments it seems that there was confusion between “Asian Indians” and “American Indians”, so the citation is not relevant. To clarify we have changed Indian Health Service to United States Indian Health Service when first used in the manuscript*

**Reviewers' comments:**

Is the manuscript technically sound, and do the data support the conclusions?

The manuscript must describe a technically sound piece of scientific research with data that

supports the conclusions. Experiments must have been conducted rigorously, with

appropriate controls, replication, and sample sizes. The conclusions must be drawn

appropriately based on the data presented.

Reviewer #1: Yes

Reviewer #2: Yes

*We have mothing to add or edit here*

2. Has the statistical analysis been performed appropriately and rigorously?

Reviewer #1: I Don't Know

*As per reviewer’s comments we have performed additional statistical analyses as noted below.*

Reviewer #2: Yes

3. Have the authors made all data underlying the findings in their manuscript fully available?

The [PLOS Data policy](http://track.editorialmanager.com/CL0/http:%2F%2Fwww.plosone.org%2Fstatic%2Fpolicies.action%23sharing/1/010f019d2323f0da-b40ca215-cd0d-42b6-aebe-282ba3050751-000000/NJIWZD2g04MjozRfUwBKGj_wBh3dD6wFeKK5aqzY1c4=256) requires authors to make all data underlying the findings described in their manuscript fully available without restriction, with rare exception (please refer to the Data Availability Statement in the manuscript PDF file). The data should be provided as part of the manuscript or its supporting information, or deposited to a public repository. For example, in addition to summary statistics, the data points behind means, medians and variance measures should be available. If there are restrictions on publicly sharing data—e.g. participant privacy or use of data from a third party—those must be specified.

Reviewer #1: Yes

Reviewer #2: Yes

*We have nothing to add here other than we are providing the full dataset used in an Excel spreadsheet, with random IDs assigned to each patient.*

4. Is the manuscript presented in an intelligible fashion and written in standard English?

PLOS ONE does not copy edit accepted manuscripts, so the language in submitted articles must be clear, correct, and unambiguous. Any typographical or grammatical errors should be corrected at revision, so please note any specific errors here.

Reviewer #1: Yes

Reviewer #2: No

*Reviewer comments on the standards have all been addressed in the revised manuscript.*

**5. Review Comments to the Author:**

**Reviewer #1:** This study's purpose is to characterize retinopathy regression in a teleophthalmology program among patients seen in primary care clinics who had very mild or mild nonproliferative diabetic retinopathy at baseline. The large sample size is a clear strength, particularly for the very mild NPDR group. However, the interpretation of both progression and regression is limited by the study design, the grading, and incomplete characterization of key systemic covariates. In its current form, the manuscript has several major issues that need to be addressed.

1) Interpretation of regression is problematic in very mild NPDR

A major concern is the interpretation of retinopathy regression, especially in the very mild NPDR group. In teleophthalmology-based grading, very mild NPDR may reflect a single microaneurysm, and isolated microaneurysms can appear and disappear over relatively short time intervals. Therefore, an apparent shift from very mild NPDR to no retinopathy may reflect biologic lesion turnover or grading variability rather than true disease regression. This is particularly important because the authors emphasize that regression was more frequent than expected in the very mild NPDR group.

*While we agree with the reviewer regarding interpretation of regression from very mild NPDR, it should be noted that the DR grade in the study is the DR grade of the worst eye (line 111, 115, 283- 286). To Illustrate, if the case has MAs only in one eye and none in the other then the DR is very mild. At next FU the MAs in the one eye have regressed but now MAs appear in the contralateral eye. The grade in this case would still be very mild and would be classified as “No Change” for analysis purposes. Regression would be classified only if MAs were absent in both eyes.*

*We also addressed potential grader grading inconsistency as a factor. In this case to we performed an identical analysis on those patients who had the same diagnoses for two consecutive FU exams. We found that the proportions for the DR outcomes and their associations with the background clinical metrics were very similar.* *This is addressed in line 286 to 295 of the revised manuscript*

2) The claim that regression was more frequent than expected is not clearly supported

Several of the studies cited in Table 1 reportedly show similar or even higher rates of regression, while others show lower rates. Based on the information presented, it is not clear that this cohort truly had high regression rates. Moreover, rates of apparent progression and regression are strongly influenced by multiple factors including but not limited to duration of diabetes, imaging method, grading, and interval between examinations. Without these data, it is difficult to conclude that regression was unusually frequent.

*First, we believe there was a misinterpretation of Table 1. Only 2 papers that quoted both regression and progression showed regression greater than progression, thus our results were ‘unexpected’. Specifically, the Kohner paper in Table 1 from UKPDS shows “30% to 50% regression with 1-3 MAs at entry and less than 20% with 4 to 5 MAs (so these are very mild for us). MAs greater than 5 related to DR progression.” Also, the Zavrelova paper stated: “4.9% regressed (HbA1c of 7.8%) and 4.0% progressed (A1c of 8.4%) with a 5-year duration of diabetes. Regression was associated with lipid medication but progression was related to HbA1cs closer to 8%. The publication by Song reported results that were like ours on regression and progression, but they did not measure HbA1c and average age of their patients was higher than that of our population. Thus, the data from our study indicate that the higher rates of regression seen here were not expected.*

*We agree that DR regression or progression can be influenced by factors not available to us for this study; however, we did note that longer diabetes durations were associated with lower regression rates.*

*The interval between FU grades has been noted and the average time between baseline and final FU visit was 22.8 ± 7.9 months (line 96).*

3) Selection bias due to incomplete follow-up is a major limitation

Roughly half of the baseline cohort did not undergo a follow-up examination within 3 years. Patients lost to follow-up in diabetic eye screening programs are often at higher risk of progression and less likely to demonstrate apparent regression. As a result, the analytic cohort may be biased toward healthier, more adherent patients with better systemic control and lower risk of worsening retinopathy and possibly more likely to show regression.

*We agree that this is one possibility. This possibility is what drove the first analysis presented in the paper, comparing patients who had a follow up exam with those who did not, as shown in lines 151 – 157 and Table 2. We observed that these two patient groups were very similar. Even when p values met the threshold for statistical significance, the percentages and means of the 2 groups were close and not meaningfully different. Further, aa second possibility is that the IHS patients were going to primary care, which is where they then receive the DR exam, when they have problems, which would result in a different type of bias from what the reviewer is concerned about. These issues are noted in lines 308 - 313 of the Discussion section.*

4) Important systemic covariates are missing

The analyses focus on diabetes therapy, duration, and A1c, but several other systemic factors relevant to retinopathy including blood pressure, lipid levels, lipid-lowering medications, renal disease are not assessed. The absence of these variables limits interpretation of the associations presented.

*We agree that the unavailability of these covariates is a limitation of the study and it is acknowledged as a study limitation in lines 305 - 308.*

It is also important to clarify whether the cohort included both type 1 and type 2 diabetes. If both were included, the proportions should be reported and analyses should account for diabetes type. If only one type was included, that should be stated explicitly.

*There were only type 2 diabetes patients in the study (line 73 - 74)*

5) Need for adjusted analyses

The authors should perform multivariable analyses evaluating whether differences in outcomes persist after adjustment for at least baseline A1c, diabetes duration, age, and other available covariates. As currently presented, the tables are suggestive but do not further examine whether these factors independently explain the outcome distributions.

*At the reviewer’s excellent suggestion, we performed multivariable logistic regression analyses and the results are summarized in Table 5 and lines 201 to 210. In addition to the multivariable regression analysis, we also calculated predicted probabilities from the new multivariable regression models, Table 6 and lines 211 to 218. We believe this facilitates interpretation of what the statistical associations practically mean.*

6) Exclusion of ungradable images needs to be quantified and discussed

The manuscript excludes ungradable images, but the number and proportion of excluded images are not clear. This is important because ungradable imaging is often non-random and may be associated with older age, media opacity, poorer health status, and potentially more advanced eye disease. Excluding such cases without quantifying them may introduce additional selection bias.

*To clarify this issue, we further characterized patients who had ungradable images at follow up both for* *the very mild NPDR cohort (6%) and for the mild NPDR cohort (9%) which is comparable to the whole cohort (7%) (Table 2). Please see the Discussion section, lines 296 -304. The analyses found no meaningful differences in the background characteristics of those patients whose images could be graded at FU vs those patients whose images could not be graded at FU.*

7) Handling of more advanced retinopathy needs clarification

The manuscript excludes diabetic macular edema and proliferative diabetic retinopathy, but

it is unclear what happened to patients with moderate or severe NPDR. Were such eyes

absent at baseline by design, or did some patients progress into those categories during

follow-up? If progression beyond mild NPDR occurred, those transitions should be clearly

described. As written, the clinical trajectory beyond the baseline categories is somewhat

difficult to follow.

*The focus of this paper is regression of NPDR among people with only very mild and mild NPDR at baseline, thus eyes with DR more advanced than mild NPDR at baseline were excluded. Progression is important for context (i.e., how many more people regressed than progressed?), but it is not the focus as prevalence and incidence of more advanced DR has been described for this population previously (References 10 and 11) and has been covered extensively in the literature.*

*The data did allow characterization of cases where progression to more advanced levels of DR were noted. This is summarized in Table 3 and Table 4.*

*Also, regression in patients with higher levels of NPDR at baseline could not be cleanly examined in this study because these patients tend to be referred out of the IHS teleophthalmology program to face-to-fact clinical care with an ophthalmologist (or, in some cases, an optometrist).*

Minor comment: because this is a telehealth screening cohort using medical record data, the limitations of such studies including the interpretation of results should be discussed more explicitly.

*This has been addressed as a study limitation in the Discussion section in lines 305 to 308.*

*All medical record data across the whole cohort was unavailable due to technology infrastructure issues. Thus, if additional medical record data were available, then the multivariable analysis would increase in scope. However, using an example of hypertension based on the SDPI report (see reference 33, “Fact Sheets and Publications” we note that approximately 65% of the IHS patients fall into the hypertensive range (>130/>80) which would favor progression rather than regression. Thus, while additional medical record data would be important, we do not believe that it would markedly change the interpretation of our results, namely that regression is more frequent than progression in the early stages of DR in this population.*

**Reviewer #2:** Overview: The authors have reported their retrograde longitudinal study on the regression of very mild and mild nonproliferative diabetic retinopathy (NPDR) in 3-year follow up. They found that 47% of very mild and 36% of mild NPDR showed regression. They associated that shorter diabetes duration, dietary modification, oral medication therapies, and lower A1c with regression in patients with very mild NPDR at baseline but not patients with mild NPDR.
This is an interesting study with relevant findings. But currently there are issues with the manuscript. Please see the critics and specific comments and address them appropriately to make it acceptable.
Critics: The language or writing is poor, needs improvement. I see improper writing in almost every sentence. The manuscript must be revised significantly to make it publishable.

Specific comments:
Title: Re-write the title. You can write: Rates of Nonproliferative Diabetic Retinopathy regression among patients in an Indian Health Service teleophthalmology program, 2015 – 2020.

*Changed as suggested.*

Abstract
I advise you to structure the abstract:

*PLOS ONE manuscript style guidelines recommend an unstructured abstract which is what we provided.*

L19: You do not have to write “(reduction in DR level)”. Remove it.
L21: Write DR, not retinopathy.
L24: Write: HbA1c with full form in its first mention and abbreviate it. Secondly, what do you mean by “for context” here? You may remove it.
L26: Correct this: “…follow up (FU) follow exams…”.

*The above changes have been implemented.*

L27: “Patients (N=2348) with very mild NPDR and patients with Mild NPDR (N=705) were included in the study.” I think the first number, (N=2348), should be written after mild NPDR.

*See lines 26 and 27*

L29: Did you also exclude moderate and severe NPDR?

*Yes; moderate and severe NPFR at baseline were excluded as this level of DR generally resulted in referral to specialty ophthalmological services so were lost to Follow up. See response to reviewer 1 comment #7*

L31: A1c is not the proper way of writing. It should be HbA1c after abbreviating glycated haemoglobin I the first mention.

*Changed throughout*

LL30, 31: Write the demographic data together – 55% female should come together with mean age of presentation.

*See line 28*

L32: very mild or mild alone is not sufficient. Write very mild NPDR or mild NPDR.

*Changed*

L34: What do you mean by “diet only”? You may write dietary modification or dietary restriction.

*This has been expanded for clarification see line 120 to 122*

Q. Where are the keywords?

*These were entered into the submission per instructions. Key words entered are as follows: “diabetic retinopathy; primary care based teleretinal assessment; regression of early stage non proliferative diabetic retinopathy”*

Introduction
L48: Before writing on regression of DR, it is important for you to highlight the magnitude or prevalence of very mild and mild NPDR compared to moderate, severe NPDR and PDR.

*Reference 10 and 11 are prior studies on the same population and summarized in lines 64 to 68*

You may also compare this in developing vs developed countries. For example, in a developing country which is also a neighbouring country to India where this study was conducted, mild NPDR is the most common DR. Refer here: https://doi.org/10.1007/s10384-023-00989-6 .

*We wish to thank the reviewer for providing this interesting citation that highlighted the need for education and community involvement in primary eye care in low-sourced areas. However, our study was not done in India so its relevance for this paper is moot. To clarify, we have redefined the first mention of the Indian Health Service in our paper as the United States Indian Health Service (line 24)*

L49: Glycaemic levels (A1c) is not proper term. Write it properly as glycated haemoglobin (HbA1c).

*Changed*

L64,66: Write “The primary aim of this study was…..”, not is.
Methods

*Changed*

L86: Why have you chosen age of 20+years? Why not 18 years?

*The research protocol defined youngest recruitment age as 20. This was chosen as patients younger than age twenty tended to be more transitory and may be lost to follow up due to changes in their geographic locations.*

Q. Did you consider noting the systemic hypertension, anaemia, blood thinners, or other systemic morbidity? These diseases might have been the confounding factors for progression/regression in your study.

*This is a limitation of the study and is addressed as a study limitation in the Discussion section, line 305 -316.*

Q. Another point – did you take any account of if the participants were taking medications like fenofibrate which reverses the DR?

*This is mentioned in Line 265 to 267 of the Discussion section.*

Results
L138: In the results the demographic details, for example number of males vs females is missing.

*The data are in Table 2.*

Q. Can you provide the comparative outcome data on insulin vs oral treatment, which had higher rate of DR regression in addition to that provided in Table 4?

*This is in the new Table 6.*

Discussion
L212-215: Re-write this sentence. It is difficult to comprehend the message.
Also improve sentence L215-216.

*Based on Reviewer 1’s suggestion of performing multivariable logistic regression, the discussion has been significantly changed so concern about improving the sentence has been part of the rewriting of the Discussion section.*

L243: Drug names – for one you start in upper case for another in lower case. Just be consistent.

*Changed as suggested.*

6. PLOS authors have the option to publish the peer review history of their article ([what does this mean?](https://track.editorialmanager.com/CL0/https:%2F%2Fjournals.plos.org%2Fplosone%2Fs%2Feditorial-and-peer-review-process%23loc-peer-review-history/1/010f019d2323f0da-b40ca215-cd0d-42b6-aebe-282ba3050751-000000/C2x59Ng9bApHiTkTPXuZcma5wYQmQNHbDOk43HXjR1U=256)). If published, this will include your full peer review and any attached files.


If you choose “no”, your identity will remain anonymous but your review may still be made public.


**Do you want your identity to be public for this peer review?** For information about this choice, including consent withdrawal, please see our [Privacy Policy](https://track.editorialmanager.com/CL0/https:%2F%2Fwww.plos.org%2Fprivacy-policy/1/010f019d2323f0da-b40ca215-cd0d-42b6-aebe-282ba3050751-000000/vYERECs_QOV6JK9qDvbEjdZpe-0JrnBZqqfEd0P-QQM=256).

Reviewer #1: No

Reviewer #2: **Yes:** Bhim B. Rai

[NOTE: If reviewer comments were submitted as an attachment file, they will be attached to this email and accessible via the submission site. Please log into your account, locate the manuscript record, and check for the action link "View Attachments". If this link does not appear, there are no attachment files.]

To ensure your figures meet our technical requirements, please review our figure guidelines: https://journals.plos.org/plosone/s/figures

You may also use PLOS’s free figure tool, NAAS, to help you prepare publication quality figures: https://journals.plos.org/plosone/s/figures#loc-tools-for-figure-preparation.

NAAS will assess whether your figures meet our technical requirements by comparing each figure against our figure specifications.
