# Supplementary material for: Rates of nonproliferative diabetic retinopathy (NPDR) regression among patients with very mild or mild NPDR in an Indian Health Service teleophthalmology program, 2015 – 2020
Source: PLoS One. 2026 Oct 16;21(10):e0361052. doi: 10.1371/journal.pone.0361052 (PMC13674746; doi:10.1371/journal.pone.0361052)
Supplement: Supplementary file 3 [file pone.0361052.s004.docx]

**September 10, 2026**

Re: PONE-D-26-08283R3

Dear PLOS Editor(s) and Reviewers,

My co-authors and I are submitting the third revision of our manuscript, “**Rates of Nonproliferative Diabetic Retinopathy (NPDR) regression among patients with Very Mild or Mild NPDR in an Indian Health Service teleophthalmology program, 2015 - 2020**” for continued consideration and publication in *PLOS*.

**Comments to the Author**
Editorial Comments. Our responses are in bold italics immediately following the comments
a) If there are ethical or legal restrictions on sharing a de-identified data set, please explain them in detail (e.g., data contain potentially identifying or sensitive patient information, data are owned by a third-party organization, etc.) and who has imposed them (e.g., a Research Ethics Committee or Institutional Review Board, etc.). Please also provide contact information for a data access committee, ethics committee, or other institutional body to which data requests may be sent.

***There are no legal or ethical restrictions for sharing the de-identified data set.***

b) If there are no restrictions, please upload the minimal anonymized data set necessary to replicate your study findings to a stable, public repository and provide us with the relevant URLs, DOIs, or accession numbers. Please see <http://www.bmj.com/content/340/bmj.c181.long> for guidelines on how to de-identify and prepare clinical data for publication. For a list of recommended repositories, please see <https://journals.plos.org/plosone/s/recommended-repositories>. You also have the option of uploading the data as Supporting Information files, but we would recommend depositing data directly to a data repository if possible.

***In the text box associated with “Data Availability” of the “Additional Information” section of the submission we have entered the link to the publicly available data set in the Harvard Dataverse repository as*** <https://doi.org/10.7910/DVN/Q5HFML>
