# Supplementary material for: Rates of nonproliferative diabetic retinopathy (NPDR) regression among patients with very mild or mild NPDR in an Indian Health Service teleophthalmology program, 2015 – 2020
Source: PLoS One. 2026 Oct 16;21(10):e0361052. doi: 10.1371/journal.pone.0361052 (PMC13674746; doi:10.1371/journal.pone.0361052)
Supplement: Peer review history

# Peer review: Decision Letter 0

**DOI:** 10.1371/journal.pone.0361052.r001

**Authors:** Yalong Dang

24 Mar 2026

Dear Dr. Bursell,

Thank you for submitting your manuscript to PLOS ONE. After careful consideration, we feel that it has merit but does not fully meet PLOS ONE’s publication criteria as it currently stands. Therefore, we invite you to submit a revised version of the manuscript that addresses the points raised during the review process.

Please submit your revised manuscript by May 08 2026 11:59PM. If you will need more time than this to complete your revisions, please reply to this message or contact the journal office at plosone@plos.org. When you're ready to submit your revision, log on to https://www.editorialmanager.com/pone/ and select the 'Submissions Needing Revision' folder to locate your manuscript file.

- A letter that responds to each point raised by the academic editor and reviewer(s). You should upload this letter as a separate file labeled 'Response to Reviewers'.
- A marked-up copy of your manuscript that highlights changes made to the original version. You should upload this as a separate file labeled 'Revised Manuscript with Track Changes'.
- An unmarked version of your revised paper without tracked changes. You should upload this as a separate file labeled 'Manuscript'.

If you would like to make changes to your financial disclosure, please include your updated statement in your cover letter. Guidelines for resubmitting your figure files are available below the reviewer comments at the end of this letter.

If applicable, we recommend that you deposit your laboratory protocols in protocols.io to enhance the reproducibility of your results. Protocols.io assigns your protocol its own identifier (DOI) so that it can be cited independently in the future. For instructions see: https://journals.plos.org/plosone/s/submission-guidelines#loc-laboratory-protocols. Additionally, PLOS ONE offers an option for publishing peer-reviewed Lab Protocol articles, which describe protocols hosted on protocols.io. Read more information on sharing protocols at https://plos.org/protocols?utm_medium=editorial-email&utm_source=authorletters&utm_campaign=protocols.

As the corresponding author, your ORCID iD is verified in the submission system and will appear in the published article. PLOS supports the use of ORCID, and we encourage all coauthors to register for an ORCID iD and use it as well. Please encourage your coauthors to verify their ORCID iD within the submission system before final acceptance, as unverified ORCID iDs will not appear in the published article. *Only*  the individual author can complete the verification step; PLOS staff *cannot*  verify ORCID iDs on behalf of authors.

We look forward to receiving your revised manuscript.

Kind regards,

Yalong Dang

Academic Editor

PLOS One

**Journal Requirements:**

1. When submitting your revision, we need you to address these additional requirements.

Please ensure that your manuscript meets PLOS ONE's style requirements, including those for file naming. The PLOS ONE style templates can be found at

https://journals.plos.org/plosone/s/file?id=wjVg/PLOSOne_formatting_sample_main_body.pdf and

https://journals.plos.org/plosone/s/file?id=ba62/PLOSOne_formatting_sample_title_authors_affiliations.pdf

2. Thank you for stating the following in the Acknowledgments Section of your manuscript:

This material is the result of work supported with resources and the use of facilities at the Indian Health Service Teleophthalmology Program, Joslin Vision Network, located in the Phoenix Area of the Indian Health Service. The contents of this article do not represent the views of the Indian Health Service or the United States Government.

We note that you have provided funding information that is not currently declared in your Funding Statement. However, funding information should not appear in the Acknowledgments section or other areas of your manuscript. We will only publish funding information present in the Funding Statement section of the online submission form.

Please remove any funding-related text from the manuscript and let us know how you would like to update your Funding Statement. Currently, your Funding Statement reads as follows:

The author(s) received no specific funding for this work.

Please include your amended statements within your cover letter; we will change the online submission form on your behalf.

3. Thank you for stating the following in the Competing Interests section:

I have read the journal's policy and the authors of this manuscript have the following competing interests:

Sven-Erik Bursell: Scientific consultant for SocialEyes Corp. and Research Consultant subcontracted by the Indian Health Service Joslin Vision Network to manage and analyze the data collected for the program.

Sterphanie J. Fonda: Subcontracted by the Indian Health Service Joslin Vision Network to manage and analyze the data collected for the program as Estenda Solutions Vice President of Research Initiatives.

Jef Stainback: Subcontracted by the Indian Health Service Joslin Vision Network to manage and analyze the data collected for the program as Estenda Solutions Quality Assurance Manager.

Dara Shahon: Employee of the Indian Health Service as Director, IHS-JVN Teleophthalmology Program at the Phoenix Indian Medical Center.

We note that one or more of the authors are employed by a commercial company.

a. Please provide an amended Funding Statement declaring this commercial affiliation, as well as a statement regarding the Role of Funders in your study. If the funding organization did not play a role in the study design, data collection and analysis, decision to publish, or preparation of the manuscript and only provided financial support in the form of authors' salaries and/or research materials, please review your statements relating to the author contributions, and ensure you have specifically and accurately indicated the role(s) that these authors had in your study. You can update author roles in the Author Contributions section of the online submission form.

Please also include the following statement within your amended Funding Statement.

“The funder provided support in the form of salaries for authors, but did not have any additional role in the study design, data collection and analysis, decision to publish, or preparation of the manuscript. The specific roles of these authors are articulated in the ‘author contributions’ section.”

If your commercial affiliation did play a role in your study, please state and explain this role within your updated Funding Statement.

b. Please also provide an updated Competing Interests Statement declaring this commercial affiliation along with any other relevant declarations relating to employment, consultancy, patents, products in development, or marketed products, etc.

Within your Competing Interests Statement, please confirm that this commercial affiliation does not alter your adherence to all PLOS ONE policies on sharing data and materials by including the following statement: "This does not alter our adherence to  PLOS ONE policies on sharing data and materials.” (as detailed online in our guide for authors http://journals.plos.org/plosone/s/competing-interests) . If this adherence statement is not accurate and  there are restrictions on sharing of data and/or materials, please state these. Please note that we cannot proceed with consideration of your article until this information has been declared.

Please include both an updated Funding Statement and Competing Interests Statement in your cover letter. We will change the online submission form on your behalf.

4. Thank you for stating the following in the Competing Interests section:

I have read the journal's policy and the authors of this manuscript have the following competing interests:

Sven-Erik Bursell: Scientific consultant for SocialEyes Corp. and Research Consultant subcontracted by the Indian Health Service Joslin Vision Network to manage and analyze the data collected for the program.

Sterphanie J. Fonda: Subcontracted by the Indian Health Service Joslin Vision Network to manage and analyze the data collected for the program as Estenda Solutions Vice President of Research Initiatives.

Jef Stainback: Subcontracted by the Indian Health Service Joslin Vision Network to manage and analyze the data collected for the program as Estenda Solutions Quality Assurance Manager.

Dara Shahon: Employee of the Indian Health Service as Director, IHS-JVN Teleophthalmology Program at the Phoenix Indian Medical Center.

Please confirm that this does not alter your adherence to all PLOS ONE policies on sharing data and materials, by including the following statement: "This does not alter our adherence to  PLOS ONE policies on sharing data and materials.” (as detailed online in our guide for authors http://journals.plos.org/plosone/s/competing-interests).  If there are restrictions on sharing of data and/or materials, please state these. Please note that we cannot proceed with consideration of your article until this information has been declared.

Please include your updated Competing Interests statement in your cover letter; we will change the online submission form on your behalf.

5. We note that you have indicated that there are restrictions to data sharing for this study. For studies involving human research participant data or other sensitive data, we encourage authors to share de-identified or anonymized data. However, when data cannot be publicly shared for ethical reasons, we allow authors to make their data sets available upon request. For information on unacceptable data access restrictions, please see http://journals.plos.org/plosone/s/data-availability#loc-unacceptable-data-access-restrictions.

Before we proceed with your manuscript, please address the following prompts:

a) If there are ethical or legal restrictions on sharing a de-identified data set, please explain them in detail (e.g., data contain potentially identifying or sensitive patient information, data are owned by a third-party organization, etc.) and who has imposed them (e.g., a Research Ethics Committee or Institutional Review Board, etc.). Please also provide contact information for a data access committee, ethics committee, or other institutional body to which data requests may be sent.

b) If there are no restrictions, please upload the minimal anonymized data set necessary to replicate your study findings to a stable, public repository and provide us with the relevant URLs, DOIs, or accession numbers. Please see http://www.bmj.com/content/340/bmj.c181.long for guidelines on how to de-identify and prepare clinical data for publication. For a list of recommended repositories, please see https://journals.plos.org/plosone/s/recommended-repositories. You also have the option of uploading the data as Supporting Information files, but we would recommend depositing data directly to a data repository if possible.

Please update your Data Availability statement in the submission form accordingly.

6. When completing the data availability statement of the submission form, you indicated that you will make your data available on acceptance. We strongly recommend all authors decide on a data sharing plan before acceptance, as the process can be lengthy and hold up publication timelines. Please note that, though access restrictions are acceptable now, your entire data will need to be made freely accessible if your manuscript is accepted for publication. This policy applies to all data except where public deposition would breach compliance with the protocol approved by your research ethics board. If you are unable to adhere to our open data policy, please kindly revise your statement to explain your reasoning and we will seek the editor's input on an exemption. Please be assured that, once you have provided your new statement, the assessment of your exemption will not hold up the peer review process.

7. If the reviewer comments include a recommendation to cite specific previously published works, please review and evaluate these publications to determine whether they are relevant and should be cited. There is no requirement to cite these works unless the editor has indicated otherwise.

[Note: HTML markup is below. Please do not edit.]

Reviewers' comments:

**Comments to the Author**

1. Is the manuscript technically sound, and do the data support the conclusions?

Reviewer #1: Yes

Reviewer #2: Yes

**********

2. Has the statistical analysis been performed appropriately and rigorously?

Reviewer #1: I Don't Know

Reviewer #2: Yes

**********

3. Have the authors made all data underlying the findings in their manuscript fully available?

The PLOS Data policy

Reviewer #1: Yes

Reviewer #2: Yes

**********

4. Is the manuscript presented in an intelligible fashion and written in standard English?

Reviewer #1: Yes

Reviewer #2: No

**********

**Reviewer #1:**  This study's purpose is to characterize retinopathy regression in a teleophthalmology program among patients seen in primary care clinics who had very mild or mild nonproliferative diabetic retinopathy at baseline. The large sample size is a clear strength, particularly for the very mild NPDR group. However, the interpretation of both progression and regression is limited by the study design, the grading, and incomplete characterization of key systemic covariates. In its current form, the manuscript has several major issues that need to be addressed.

1) Interpretation of regression is problematic in very mild NPDR

A major concern is the interpretation of retinopathy regression, especially in the very mild NPDR group. In teleophthalmology-based grading, very mild NPDR may reflect a single microaneurysm, and isolated microaneurysms can appear and disappear over relatively short time intervals. Therefore, an apparent shift from very mild NPDR to no retinopathy may reflect biologic lesion turnover or grading variability rather than true disease regression. This is particularly important because the authors emphasize that regression was more frequent than expected in the very mild NPDR group.

2) The claim that regression was more frequent than expected is not clearly supported

Several of the studies cited in Table 1 reportedly show similar or even higher rates of regression, while others show lower rates. Based on the information presented, it is not clear that this cohort truly had high regression rates. Moreover, rates of apparent progression and regression are strongly influenced by multiple factors including but not limited to duration of diabetes, imaging method, grading, and interval between examinations. Without these data, it is difficult to conclude that regression was unusually frequent.

3) Selection bias due to incomplete follow-up is a major limitation

Roughly half of the baseline cohort did not undergo a follow-up examination within 3 years. Patients lost to follow-up in diabetic eye screening programs are often at higher risk of progression and less likely to demonstrate apparent regression. As a result, the analytic cohort may be biased toward healthier, more adherent patients with better systemic control and lower risk of worsening retinopathy and possibly more likely to show regression.

4) Important systemic covariates are missing

The analyses focus on diabetes therapy, duration, and A1c, but several other systemic factors relevant to retinopathy including blood pressure, lipid levels, lipid-lowering medications, renal disease are not assessed. The absence of these variables limits interpretation of the associations presented.

It is also important to clarify whether the cohort included both type 1 and type 2 diabetes. If both were included, the proportions should be reported and analyses should account for diabetes type. If only one type was included, that should be stated explicitly.

5) Need for adjusted analyses

The authors should perform multivariable analyses evaluating whether differences in outcomes persist after adjustment for at least baseline A1c, diabetes duration, age, and other available covariates. As currently presented, the tables are suggestive but do not further examine whether these factors independently explain the outcome distributions.

6) Exclusion of ungradable images needs to be quantified and discussed

The manuscript excludes ungradable images, but the number and proportion of excluded images are not clear. This is important because ungradable imaging is often non-random and may be associated with older age, media opacity, poorer health status, and potentially more advanced eye disease. Excluding such cases without quantifying them may introduce additional selection bias.

7) Handling of more advanced retinopathy needs clarification

The manuscript excludes diabetic macular edema and proliferative diabetic retinopathy, but it is unclear what happened to patients with moderate or severe NPDR. Were such eyes absent at baseline by design, or did some patients progress into those categories during follow-up? If progression beyond mild NPDR occurred, those transitions should be clearly described. As written, the clinical trajectory beyond the baseline categories is somewhat difficult to follow.

Minor comment: because this is a telehealth screening cohort using medical record data, the limitations of such studies including the interpretation of results should be discussed more explicitly.

**Reviewer #2:**  Overview: The authors have reported their retrograde longitudinal study on the regression of very mild and mild nonproliferative diabetic retinopathy (NPDR) in 3-year follow up. They found that 47% of very mild and 36% of mild NPDR showed regression. They associated that shorter diabetes duration, dietary modification, oral medication therapies, and lower A1c with regression in patients with very mild NPDR at baseline but not patients with mild NPDR.

This is an interesting study with relevant findings. But currently there are issues with the manuscript. Please see the critics and specific comments and address them appropriately to make it acceptable.

Critics: The language or writing is poor, needs improvement. I see improper writing in almost every sentence. The manuscript must be revised significantly to make it publishable.

Specific comments:

Title: Re-write the title. You can write: Rates of Nonproliferative Diabetic Retinopathy regression among patients in an Indian Health Service teleophthalmology program, 2015 – 2020.

Abstract

I advise you to structure the abstract:

L19: You do not have to write “(reduction in DR level)”. Remove it.

L21: Write DR, not retinopathy.

L24: Write: HbA1c with full form in its first mention and abbreviate it. Secondly, what do you mean by “for context” here? You may remove it.

L26: Correct this: “…follow up (FU) follow exams…”.

L27: “Patients (N=2348) with very mild NPDR and patients with Mild NPDR (N=705) were included in the study.” I think the first number, (N=2348), should be written after mild NPDR.

L29: Did you also exclude moderate and severe NPDR?

L31: A1c is not the proper way of writing. It should be HbA1c after abbreviating glycated haemoglobin I the first mention.

LL30, 31: Write the demographic data together – 55% female should come together with mean age of presentation.

L32: very mild or mild alone is not sufficient. Write very mild NPDR or mild NPDR.

L34: What do you mean by “diet only”? You may write dietary modification or dietary restriction.

Q. Where are the keywords?

Introduction

L48: Before writing on regression of DR, it is important for you to highlight the magnitude or prevalence of very mild and mild NPDR compared to moderate, severe NPDR and PDR. You may also compare this in developing vs developed countries. For example, in a developing country which is also a neighbouring country to India where this study was conducted, mild NPDR is the most common DR. Refer here: https://doi.org/10.1007/s10384-023-00989-6 .

L49: Glycaemic levels (A1c) is not proper term. Write it properly as glycated haemoglobin (HbA1c).

L64,66: Write “The primary aim of this study was…..”, not is.

Methods

L86: Why have you chosen age of 20+years? Why not 18 years?

Q. Did you consider noting the systemic hypertension, anaemia, blood thinners, or other systemic morbidity? These diseases might have been the confounding factors for progression/regression in your study.

Q. Another point – did you take any account of if the participants were taking medications like fenofibrate which reverses the DR?

Results

L138: In the results the demographic details, for example number of males vs females is missing.

Q. Can you provide the comparative outcome data on insulin vs oral treatment, which had higher rate of DR regression in addition to that provided in Table 4?

Discussion

L212-215: Re-write this sentence. It is difficult to comprehend the message.

Also improve sentence L215-216.

L243: Drug names – for one you start in upper case for another in lower case. Just be consistent.

**********

what does this mean?). If published, this will include your full peer review and any attached files.

If you choose “no”, your identity will remain anonymous but your review may still be made public.

**Do you want your identity to be public for this peer review?**  For information about this choice, including consent withdrawal, please see our Privacy Policy

Reviewer #1: No

Reviewer #2: **Yes:**  Bhim B. Rai

**********

[NOTE: If reviewer comments were submitted as an attachment file, they will be attached to this email and accessible via the submission site. Please log into your account, locate the manuscript record, and check for the action link "View Attachments". If this link does not appear, there are no attachment files.]

To ensure your figures meet our technical requirements, please review our figure guidelines: https://journals.plos.org/plosone/s/figures

You may also use PLOS’s free figure tool, NAAS, to help you prepare publication quality figures: https://journals.plos.org/plosone/s/figures#loc-tools-for-figure-preparation.

NAAS will assess whether your figures meet our technical requirements by comparing each figure against our figure specifications.

# Author comment: Author response to Decision Letter 1

**DOI:** 10.1371/journal.pone.0361052.r002

1 Jun 2026

Summary of changes:

In response to comments from Reviewer 1 regarding the need for a multivariable logistic regression analysis, we performed the suggested analysis presented the results in a new table (Table 5). To ground these results, we additionally calculated model-based predicted probabilities in presented these in another new table (Table 6). This latter table illustrates how the probability of DR regression decreased progressively with increasing HbA1c, by diabetes treatment categories. Due to the multivariable logistic regressions, the p-value columns in Table 3 and Table 4 from previous bivariate analyses became redundant and have been deleted.

In the original version of Table 3, we combined the outcome of “any progression from very mild NPDR” as a single group. Given reviewer comments, it seemed important to differentiate between “progression from very mild NPDR to mild NPDR” and “progression from very mild NPDR to DR levels greater than mild NPDR”. This differentiation is now shown in the revised Table 3.

The underlying data base has been published in the Harvard Dataverse repository and is publicly available at the following link: https://doi.org/10.7910/DVN/Q5HFML

Upload to protocols.io if applicable:

This is not applicable to this manuscript

ORCID ID Numbers

Completed for all authors who have ORCID ID numbers

Journal Requirements:

1. When submitting your revision, we need you to address these additional requirements.

PLOS ONE's style/format requirements have been implemented as outlined in the PLOS ONE style templates.

2. Thank you for stating the following in the Acknowledgments Section of your manuscript:

This material is the result of work supported with resources and the use of facilities at the Indian Health Service Teleophthalmology Program, Joslin Vision Network, located in the Phoenix Area of the Indian Health Service. The contents of this article do not represent the views of the Indian Health Service or the United States Government.

The Acknowledgement Section is amended to read as follows “The content of this article does not represent the views of the Indian Health Service or the United States Government.”

Amended Funding Statement as follows: “The funder, Estenda Solutions, a commercial company, provided support in the form of author salaries and research materials but did not have any additional role in the study design, data collection and analysis, decision to publish, or preparation of the manuscript. The specific roles of these authors are articulated in the ‘author contributions’ section.”

3. Competing Interests section.

Author Bursell declared SocialEyes Corp as a potential competing interest. As this consulting position is unpaid with no other financial associations it does not need to be declared here so it has been removed from the Competing Interests section.

Updated Competing Interests statement:

I have read the journal's policy and the authors of this manuscript have the following competing interests:

Sven-Erik Bursell: Estenda Solutions Research Consultant subcontracted by the Indian Health Service Joslin Vision Network to manage and analyze the data collected for the program.

Stephanie J. Fonda: Subcontracted by the Indian Health Service Joslin Vision Network to manage and analyze the data collected for the program as Estenda Solutions Vice President of Research Initiatives.

Jeff Stainback: Subcontracted by the Indian Health Service Joslin Vision Network to manage and analyze the data collected for the program as Estenda Solutions Quality Assurance Manager.

Dara Shahon: Employee of the Indian Health Service as Director, IHS-JVN Teleophthalmology Program at the Phoenix Indian Medical Center.

This does not alter our adherence to PLOS ONE policies on sharing data and materials.

5. We note that you have indicated that there are restrictions to data sharing for this study.

These restrictions have been resolved and through the IHS National IRB committee and the Phoenix Area IHS IRB committee we have received approval to publicly share the anonymized underlying data associated with this study.

Thus, there are no longer any restrictions on data sharing and our Data Availability statement in the submission form will be updated accordingly.

7. Reviewer comments include a recommendation to cite specific previously published work.

Reviewer 2 recommended citation of a DR study from Bhutan (https://doi.org/10.1007/s10384-023-00989-6) that documented DR prevalence and severity but not regression. Based on Reviewer 2 comments it seems that there was confusion between “Asian Indians” and “American Indians”, so the citation is not relevant. To clarify we have changed Indian Health Service to United States Indian Health Service when first used in the manuscript

Reviewers' comments:

Is the manuscript technically sound, and do the data support the conclusions?

The manuscript must describe a technically sound piece of scientific research with data that

supports the conclusions. Experiments must have been conducted rigorously, with

appropriate controls, replication, and sample sizes. The conclusions must be drawn

appropriately based on the data presented.

Reviewer #1: Yes

Reviewer #2: Yes

We have mothing to add or edit here

________________________________________

2. Has the statistical analysis been performed appropriately and rigorously?

Reviewer #1: I Don't Know

As per reviewer’s comments we have performed additional statistical analyses as noted below.

Reviewer #2: Yes

________________________________________

3. Have the authors made all data underlying the findings in their manuscript fully available?

The PLOS Data policy requires authors to make all data underlying the findings described in their manuscript fully available without restriction, with rare exception (please refer to the Data Availability Statement in the manuscript PDF file). The data should be provided as part of the manuscript or its supporting information, or deposited to a public repository. For example, in addition to summary statistics, the data points behind means, medians and variance measures should be available. If there are restrictions on publicly sharing data—e.g. participant privacy or use of data from a third party—those must be specified.

Reviewer #1: Yes

Reviewer #2: Yes

We have nothing to add here other than we are providing the full dataset used in an Excel spreadsheet, with random IDs assigned to each patient.

________________________________________

4. Is the manuscript presented in an intelligible fashion and written in standard English?

PLOS ONE does not copy edit accepted manuscripts, so the language in submitted articles must be clear, correct, and unambiguous. Any typographical or grammatical errors should be corrected at revision, so please note any specific errors here.

Reviewer #1: Yes

Reviewer #2: No

Reviewer comments on the standards have all been addressed in the revised manuscript.

________________________________________

5. Review Comments to the Author:

Reviewer #1: This study's purpose is to characterize retinopathy regression in a teleophthalmology program among patients seen in primary care clinics who had very mild or mild nonproliferative diabetic retinopathy at baseline. The large sample size is a clear strength, particularly for the very mild NPDR group. However, the interpretation of both progression and regression is limited by the study design, the grading, and incomplete characterization of key systemic covariates. In its current form, the manuscript has several major issues that need to be addressed.

1) Interpretation of regression is problematic in very mild NPDR

A major concern is the interpretation of retinopathy regression, especially in the very mild NPDR group. In teleophthalmology-based grading, very mild NPDR may reflect a single microaneurysm, and isolated microaneurysms can appear and disappear over relatively short time intervals. Therefore, an apparent shift from very mild NPDR to no retinopathy may reflect biologic lesion turnover or grading variability rather than true disease regression. This is particularly important because the authors emphasize that regression was more frequent than expected in the very mild NPDR group.

While we agree with the reviewer regarding interpretation of regression from very mild NPDR, it should be noted that the DR grade in the study is the DR grade of the worst eye (line 111, 115, 283- 286). To Illustrate, if the case has MAs only in one eye and none in the other then the DR is very mild. At next FU the MAs in the one eye have regressed but now MAs appear in the contralateral eye. The grade in this case would still be very mild and would be classified as “No Change” for analysis purposes. Regression would be classified only if MAs were absent in both eyes.

We also addressed potential grader grading inconsistency as a factor. In this case to we performed an identical analysis on those patients who had the same diagnoses for two consecutive FU exams. We found that the proportions for the DR outcomes and their associations with the background clinical metrics were very similar. This is addressed in line 286 to 295 of the revised manuscript

2) The claim that regression was more frequent than expected is not clearly supported

Several of the studies cited in Table 1 reportedly show similar or even higher rates of regression, while others show lower rates. Based on the information presented, it is not clear that this cohort truly had high regression rates. Moreover, rates of apparent progression and regression are strongly influenced by multiple factors including but not limited to duration of diabetes, imaging method, grading, and interval between examinations. Without these data, it is difficult to conclude that regression was unusually frequent.

First, we believe there was a misinterpretation of Table 1. Only 2 papers that quoted both regression and progression showed regression greater than progression, thus our results were ‘unexpected’. Specifically, the Kohner paper in Table 1 from UKPDS shows “30% to 50% regression with 1-3 MAs at entry and less than 20% with 4 to 5 MAs (so these are very mild for us). MAs greater than 5 related to DR progression.” Also, the Zavrelova paper stated: “4.9% regressed (HbA1c of 7.8%) and 4.0% progressed (A1c of 8.4%) with a 5-year duration of diabetes. Regression was associated with lipid medication but progression was related to HbA1cs closer to 8%. The publication by Song reported results that were like ours on regression and progression, but they did not measure HbA1c and average age of their patients was higher than that of our population. Thus, the data from our study indicate that the higher rates of regression seen here were not expected.

We agree that DR regression or progression can be influenced by factors not available to us for this study; however, we did note that longer diabetes durations were associated with lower regression rates.

The interval between FU grades has been noted and the average time between baseline and final FU visit was 22.8 + 7.9 months (line 96).

3) Selection bias due to incomplete follow-up is a major limitation

Roughly half of the baseline cohort did not undergo a follow-up examination within 3 years. Patients lost to follow-up in diabetic eye screening programs are often at higher risk of progression and less likely to demonstrate apparent regression. As a result, the analytic cohort may be biased toward healthier, more adherent patients with better systemic control and lower risk of worsening retinopathy and possibly more likely to show regression.

We agree that this is one possibility. This possibility is what drove the first analysis presented in the paper, comparing patients who had a follow up exam with those who did not, as shown in lines 151 – 157 and Table 2. We observed that these two patient groups were very similar. Even when p values met the threshold for statistical significance, the percentages and means of the 2 groups were close and not meaningfully different. Further, aa second possibility is that the IHS patients were going to primary care, which is where they then receive the DR exam, when they have problems, which would result in a different type of bias from what the reviewer is concerned about. These issues are noted in lines 308 - 313 of the Discussion section.

4) Important systemic covariates are missing

The analyses focus on diabetes therapy, duration, and A1c, but several other systemic factors relevant to retinopathy including blood pressure, lipid levels, lipid-lowering medications, renal disease are not assessed. The absence of these variables limits interpretation of the associations presented.

We agree that the unavailability of these covariates is a limitation of the study and it is acknowledged as a study limitation in lines 305 - 308.

It is also important to clarify whether the cohort included both type 1 and type 2 diabetes. If both were included, the proportions should be reported and analyses should account for diabetes type. If only one type was included, that should be stated explicitly.

There were only type 2 diabetes patients in the study (line 73 - 74)

5) Need for adjusted analyses

The authors should perform multivariable analyses evaluating whether differences in outcomes persist after adjustment for at least baseline A1c, diabetes duration, age, and other available covariates. As currently presented, the tables are suggestive but do not further examine whether these factors independently explain the outcome distributions.

At the reviewer’s excellent suggestion, we performed multivariable logistic regression analyses and the results are summarized in Table 5 and lines 201 to 210. In addition to the multivariable regression analysis, we also calculated predicted probabilities from the new multivariable regression models, Table 6 and lines 211 to 218. We believe this facilitates interpretation of what the statistical associations practically mean.

6) Exclusion of ungradable images needs to be quantified and discussed

The manuscript excludes ungradable images, but the number and proportion of excluded images are not clear. This is important because ungradable imaging is often non-random and may be associated with older age, media opacity, poorer health status, and potentially more advanced eye disease. Excluding such cases without quantifying them may introduce additional selection bias.

To clarify this issue, we further characterized patients who had ungradable images at follow up both for the very mild NPDR cohort (6%) and for the mild NPDR cohort (9%) which is comparable to the whole cohort (7%) (Table 2). Please see the Discussion section, lines 296 -304. The analyses found no meaningful differences in the background characteristics of those patients whose images could be graded at FU vs those patients whose images could not be graded at FU.

7) Handling of more advanced retinopathy needs clarification

The manuscript excludes diabetic macular edema and proliferative diabetic retinopathy, but

it is unclear what happened to patients with moderate or severe NPDR. Were such eyes

absent at baseline by design, or did some patients progress into those categories during

follow-up? If progression beyond mild NPDR occurred, those transitions should be clearly

described. As written, the clinical trajectory beyond the baseline categories is somewhat

difficult to follow.

The focus of this paper is regression of NPDR among people with only very mild and mild NPDR at baseline, thus eyes with DR more advanced than mild NPDR at baseline were excluded. Progression is important for context (i.e., how many more people regressed than progressed?), but it is not the focus as prevalence and incidence of more advanced DR has been described for this population previously (References 10 and 11) and has been covered extensively in the literature.

The data did allow characterization of cases where progression to more advanced levels of DR were noted. This is summarized in Table 3 and Table 4.

Also, regression in patients with higher levels of NPDR at baseline could not be cleanly examined in this study because these patients tend to be referred out of the IHS teleophthalmology program to face-to-fact clinical care with an ophthalmologist (or, in some cases, an optometrist).

Minor comment: because this is a telehealth screening cohort using medical record data, the limitations of such studies including the interpretation of results should be discussed more explicitly.

This has been addressed as a study limitation in the Discussion section in lines 305 to 308.

All medical record data across the whole cohort was unavailable due to technology infrastructure issues. Thus, if additional medical record data were available, then the multivariable analysis would increase in scope. However, using an example of hypertension based on the SDPI report (see reference 33, “Fact Sheets and Publications” we note that approximately 65% of the IHS patients fall into the hypertensive range (>130/>80) which would favor progression rather than regression. Thus, while additional medical record data would be important, we do not believe that it would markedly change the interpretation of our results, namely that regression is more frequent than progression in the early stages of DR in this population.

Reviewer #2: Overview: The authors have reported their retrograde longitudinal study on the regression of very mild and mild nonproliferative diabetic retinopathy (NPDR) in 3-year follow up. They found that 47% of very mild and 36% of mild NPDR showed regression. They associated that shorter diabetes duration, dietary modification, oral medication therapies, and lower A1c with regression in patients with very mild NPDR at baseline but not patients with mild NPDR.

This is an interesting study with relevant findings. But currently there are issues with the manuscript. Please see the critics and specific comments and address them appropriately to make it acceptable.

Critics: The language or writing is poor, needs improvement. I see improper writing in almost every sentence. The manuscript must be revised significantly to make it publishable.

Specific comments:

Title: Re-write the title. You can write: Rates of Nonproliferative Diabetic Retinopathy regression among patients in an Indian Health Service teleophthalmology program, 2015 – 2020.

Changed as suggested.

Abstract

I advise you to structure the abstract:

PLOS ONE manuscript style guidelines recommend an unstructured abstract which is what we provided.

L19: You do not have to write “(reduction in DR level)”. Remove it.

L21: Write DR, not retinopathy.

L24: Write: HbA1c with full form in its first mention and abbreviate it. Secondly, what do you mean by “for context” here? You may remove it.

L26: Correct this: “…follow up (FU) follow exams…”.

The above changes have been implemented.

L27: “Patients (N=2348) with very mild NPDR and patients with Mild NPDR (N=705) were included in the study.” I think the first number, (N=2348), should be written after mild NPDR.

See lines 26 and 27

L29: Did you also exclude moderate and severe NPDR?

Yes; moderate and severe NPFR at baseline were excluded as this level of DR generally resulted in referral to specialty ophthalmological services so were lost to Follow up. See response to reviewer 1 comment #7

L31: A1c is not the proper way of writing. It should be HbA1c after abbreviating glycated haemoglobin I the first mention.

Changed throughout

LL30, 31: Write the demographic data together – 55% female should come together with mean age of presentation.

See line 28

L32: very mild or mild alone is not sufficient. Write very mild NPDR or mild NPDR.

Changed

L34: What do you mean by “diet only”? You may write dietary modification or dietary restriction.

This has been expanded for clarification see line 120 to 122

Q. Where are the keywords?

These were entered into the submission per instructions. Key words entered are as follows: “diabetic retinopathy; primary care based teleretinal assessment; regression of early stage non proliferative diabetic retinopathy”

Introduction

L48: Before writing on regression of DR, it is important for you to highlight the magnitude or prevalence of very mild and mild NPDR compared to moderate, severe NPDR and PDR.

Reference 10 and 11 are prior studies on the same population and summarized in lines 64 to 68

You may also compare this in developing vs developed countries. For example, in a developing country which is also a neighbouring country to India where this study was conducted, mild NPDR is the most common DR. Refer here: https://doi.org/10.1007/s10384-023-00989-6 .

We wish to thank the reviewer for providing this interesting citation that highlighted the need for education and community involvement in primary eye care in low-sourced areas. However, our study was not done in India so its relevance for this paper is moot. To clarify, we have redefined the first mention of the Indian Health Service in our paper as the United States Indian Health Service (line 24)

L49: Glycaemic levels (A1c) is not proper term. Write it properly as glycated haemoglobin (HbA1c).

Changed

L64,66: Write “The primary aim of this study was…..”, not is.

Methods

Changed

L86: Why have you chosen age of 20+years? Why not 18 years?

The research protocol defined youngest recruitment age as 20. This was chosen as patients younger than age twenty tended to be more transitory and may be lost to follow up due to changes in their geographic locations.

Q. Did you consider noting the systemic hypertension, anaemia, blood thinners, or other systemic morbidity? These diseases might have been the confounding factors for progression/regression in your study.

This is a limitation of the study and is addressed as a study limitation in the Discussion section, line 305 -316.

Q. Another point – did you take any account of if the participants were taking medications like fenofibrate which reverses the DR?

This is mentioned in Line 265 to 267 of the Discussion section.

Results

L138: In the results the demographic details, for example number of males vs females is missing.

The data are in Table 2.

Q. Can you provide the comparative outcome data on insulin vs oral treatment, which had higher rate of DR regression in addition to that provided in Table 4?

This is in the new Table 6.

Discussion

L212-215: Re-write this sentence. It is difficult to comprehend the message.

Also improve sentence L215-216.

Based on Reviewer 1’s suggestion of performing multivariable logistic regression, the discussion has been significantly changed so concern about improving the sentence has been part of the rewriting of the Discussion section.

L243: Drug names – for one you start in upper case for another in lower case. Just be consistent.

Changed as suggested.________________________________________

6. PLOS authors have the option to publish the peer review history of their article (what does this mean?). If published, this will include your full peer review and any attached files.

If you choose “no”, your identity will remain anonymous but your review may still be made public.

Do you want your identity to be public for this peer review? For information about this choice, including consent withdrawal, please see our Privacy Policy.

Reviewer #1: No

Reviewer #2: Yes: Bhim B. Rai

________________________________________

Attachment Submitted filename: Response to Reviewers.docx

# Peer review: Decision Letter 1

**DOI:** 10.1371/journal.pone.0361052.r003

**Authors:** Yalong Dang

14 Jul 2026

Dear Dr. Bursell,

Thank you for submitting your manuscript to PLOS ONE. After careful consideration, we feel that it has merit but does not fully meet PLOS ONE’s publication criteria as it currently stands. Therefore, we invite you to submit a revised version of the manuscript that addresses the points raised during the review process.

plosone@plos.org. When you're ready to submit your revision, log on to https://www.editorialmanager.com/pone/ and select the 'Submissions Needing Revision' folder to locate your manuscript file.

- A letter that responds to each point raised by the academic editor and reviewer(s). You should upload this letter as a separate file labeled 'Response to Reviewers'.
- A marked-up copy of your manuscript that highlights changes made to the original version. You should upload this as a separate file labeled 'Revised Manuscript with Track Changes'.
- An unmarked version of your revised paper without tracked changes. You should upload this as a separate file labeled 'Manuscript'.

If you would like to make changes to your financial disclosure, please include your updated statement in your cover letter. Guidelines for resubmitting your figure files are available below the reviewer comments at the end of this letter.

If applicable, we recommend that you deposit your laboratory protocols in protocols.io to enhance the reproducibility of your results. Protocols.io assigns your protocol its own identifier (DOI) so that it can be cited independently in the future. For instructions see: https://journals.plos.org/plosone/s/submission-guidelines#loc-laboratory-protocols. Additionally, PLOS ONE offers an option for publishing peer-reviewed Lab Protocol articles, which describe protocols hosted on protocols.io. Read more information on sharing protocols at https://plos.org/protocols?utm_medium=editorial-email&utm_source=authorletters&utm_campaign=protocols.

As the corresponding author, your ORCID iD is verified in the submission system and will appear in the published article. PLOS supports the use of ORCID, and we encourage all coauthors to register for an ORCID iD and use it as well. Please encourage your coauthors to verify their ORCID iD within the submission system before final acceptance, as unverified ORCID iDs will not appear in the published article. *Only*  the individual author can complete the verification step; PLOS staff *cannot*  verify ORCID iDs on behalf of authors.

We look forward to receiving your revised manuscript.

Kind regards,

Yalong Dang

Academic Editor

PLOS One

**Journal Requirements:**

If the reviewer comments include a recommendation to cite specific previously published works, please review and evaluate these publications to determine whether they are relevant and should be cited. There is no requirement to cite these works unless the editor has indicated otherwise.

[Note: HTML markup is below. Please do not edit.]

Reviewers' comments:

Reviewer's Responses to Questions

**Comments to the Author**

Reviewer #2: (No Response)

Reviewer #3: All comments have been addressed

**********

2. Is the manuscript technically sound, and do the data support the conclusions?

Reviewer #2: Yes

Reviewer #3: Partly

**********

3. Has the statistical analysis been performed appropriately and rigorously?

Reviewer #2: Yes

Reviewer #3: No

**********

4. Have the authors made all data underlying the findings in their manuscript fully available?

The PLOS Data policy

Reviewer #2: No

Reviewer #3: Yes

**********

5. Is the manuscript presented in an intelligible fashion and written in standard English?

Reviewer #2: Yes

Reviewer #3: No

**********

Reviewer #2: The authors have addressed all my comments satisfactorily, except one point - I suggested that the authors should made a briefly present the prevalence of different severity of DR in India compared to neighboring/regional countries and other developed countries. This information is significant as the paper is on the regression of mild and very mild NPDR. The authors are advised to address this point.

Reviewer #3: The revised manuscript and the point-by-point response are exceptionally well-crafted, effectively addressing both the scientific and clinical concerns raised by the reviewers in a highly strategic manner. The addition of the multivariable logistic regression analysis, along with the model-based predicted probabilities, significantly strengthens the study's epidemiological validity and directly answers the first reviewer's primary critique. Furthermore, the clinical defense regarding microaneurysm turnover is highly persuasive; explaining the worst-eye grading criteria and backing it up with a new sensitivity analysis provides a rock-solid argument against grading variability. The response also tactfully handles the second reviewer's geographic mix-up regarding the Bhutan study, politely clearing up the confusion between American and Asian populations. However, before making the final submission, please correct a few lingering typos to ensure complete professionalism. In the response letter, fix the typo "mothing" to "nothing", change the grammatical error "in presented" to "and presented", and ensure the co-author names are spelled correctly as "Stephanie" and "Jeff" in the updated competing interests section. Within the manuscript itself, please standardize the data precision in the abstract by changing 0.2749 to 0.27 so it consistently matches the two-decimal format used throughout the rest of the paper. Additionally, remember to remove the accidental duplicate text in "contribute cont to" within the discussion section, and replace the plain text plus signs in the abstract with proper plus-minus symbols, such as 56 ± 12.8. Overall, this is a remarkably mature and thorough revision. The core scientific issues have been successfully resolved, and once these minor formatting and spelling details are polished, the manuscript will be in fantastic shape and practically ready for publication.

**********

what does this mean?). If published, this will include your full peer review and any attached files.

If you choose “no”, your identity will remain anonymous but your review may still be made public.

**Do you want your identity to be public for this peer review?**  For information about this choice, including consent withdrawal, please see our Privacy Policy

Reviewer #2: **Yes:**  Bhim Bahadur Rai

Reviewer #3: No

**********

[NOTE: If reviewer comments were submitted as an attachment file, they will be attached to this email and accessible via the submission site. Please log into your account, locate the manuscript record, and check for the action link "View Attachments". If this link does not appear, there are no attachment files.]

To ensure your figures meet our technical requirements, please review our figure guidelines: https://journals.plos.org/plosone/s/figures

You may also use PLOS’s free figure tool, NAAS, to help you prepare publication quality figures: https://journals.plos.org/plosone/s/figures#loc-tools-for-figure-preparation.

NAAS will assess whether your figures meet our technical requirements by comparing each figure against our figure specifications.

# Author comment: Author response to Decision Letter 2

**DOI:** 10.1371/journal.pone.0361052.r004

29 Jul 2026

Journal Requirements:

No revisions required for the Amended Funding Statement, Amended Competing Interest Statement as detailed in the prior revised Cover Letter.

Additionally, as noted the prior Cover Letter the underlying data is publicly available at the following link: https://doi.org/10.7910/DVN/Q5HFML

Comments to the Author

1. If the authors have adequately addressed your comments raised in a previous round of review and you feel that this manuscript is now acceptable for publication, you may indicate that here to bypass the “Comments to the Author” section, enter your conflict of interest statement in the “Confidential to Editor” section, and submit your "Accept" recommendation.

Reviewer #2: (No Response)

Reviewer #3: All comments have been addressed

We have nothing to add or edit here

________________________________________

2. Is the manuscript technically sound, and do the data support the conclusions?

The manuscript must describe a technically sound piece of scientific research with data that supports the conclusions. Experiments must have been conducted rigorously, with appropriate controls, replication, and sample sizes. The conclusions must be drawn appropriately based on the data presented.

Reviewer #2: Yes

Reviewer #3: Partly

We have nothing to add or edit here

________________________________________

3. Has the statistical analysis been performed appropriately and rigorously?

Reviewer #2: Yes

Reviewer #3: No

Confirmed with this reviewer that this response should be “Yes” rather than “No”.

________________________________________

4. Have the authors made all data underlying the findings in their manuscript fully available?

The PLOS Data policy requires authors to make all data underlying the findings described in their manuscript fully available without restriction, with rare exception (please refer to the Data Availability Statement in the manuscript PDF file). The data should be provided as part of the manuscript or its supporting information, or deposited to a public repository. For example, in addition to summary statistics, the data points behind means, medians and variance measures should be available. If there are restrictions on publicly sharing data—e.g. participant privacy or use of data from a third party—those must be specified.

Reviewer #2: No

Reviewer #3: Yes

The supporting information has been deposited in a repository, Harvard Dataverse and can be publicly accessed with the following link: https://doi.org/10.7910/DVN/Q5HFML

________________________________________

5. Is the manuscript presented in an intelligible fashion and written in standard English?

PLOS ONE does not copyedit accepted manuscripts, so the language in submitted articles must be clear, correct, and unambiguous. Any typographical or grammatical errors should be corrected at revision, so please note any specific errors here.

Reviewer #2: Yes

Reviewer #3: No

Typographical and grammatical errors noted by Reviewer 3 have been corrected. See Reviewer 3 comments below.

________________________________________

6. Review Comments to the Author

Please use the space provided to explain your answers to the questions above. You may also include additional comments for the author, including concerns about dual publication, research ethics, or publication ethics. (Please upload your review as an attachment if it exceeds 20,000 characters)

Reviewer #2: The authors have addressed all my comments satisfactorily, except one point - I suggested that the authors should made a briefly present the prevalence of different severity of DR in India compared to neighboring/regional countries and other developed countries. This information is significant as the paper is on the regression of mild and very mild NPDR. The authors are advised to address this point.

Based on reviewer’s recommendation we have added a reference to Table 1 detailing the progression and regression of DR in an Indian-based retinal data network (SN-DREAMS II). This is an appropriate reference to add as it details progression and regression which is the focus of Table 1. The added reference [36] is as follows: Raman, R, Ganesan, S, Pal, S, Gella, L, Kulothungan, V, Sharma, T. Incidence and Progression of Diabetic Retinopathy in Urban India: Sankara Nethralaya-Diabetic Retinopathy Epidemiology and Molecular Genetics Study (SN-DREAMS II), Report 1. Ophthalmic Epidemiology. 2017;24(5):294–302.

The other references in Table 1 identify other studies on DR regression and progression originating in other developed and regional countries.

Reviewer #3: The revised manuscript and the point-by-point response are exceptionally well-crafted, effectively addressing both the scientific and clinical concerns raised by the reviewers in a highly strategic manner. The addition of the multivariable logistic regression analysis, along with the model-based predicted probabilities, significantly strengthens the study's epidemiological validity and directly answers the first reviewer's primary critique. Furthermore, the clinical defense regarding microaneurysm turnover is highly persuasive; explaining the worst-eye grading criteria and backing it up with a new sensitivity analysis provides a rock-solid argument against grading variability. The response also tactfully handles the second reviewer's geographic mix-up regarding the Bhutan study, politely clearing up the confusion between American and Asian populations. However, before making the final submission, please correct a few lingering typos to ensure complete professionalism. In the response letter, fix the typo "mothing" to "nothing", change the grammatical error "in presented" to "and presented", and ensure the co-author names are spelled correctly as "Stephanie" and "Jeff" in the updated competing interests section. Within the manuscript itself, please standardize the data precision in the abstract by changing 0.2749 to 0.27 so it consistently matches the two-decimal format used throughout the rest of the paper. Additionally, remember to remove the accidental duplicate text in "contribute cont to" within the discussion section, and replace the plain text plus signs in the abstract with proper plus-minus symbols, such as 56 ± 12.8. Overall, this is a remarkably mature and thorough revision. The core scientific issues have been successfully resolved, and once these minor formatting and spelling details are polished, the manuscript will be in fantastic shape and practically ready for publication.

We would like to thank this reviewer for the favorable comments on the statistical analysis.

Grammatical error corrected

Co Authors names are spelled correctly in the updated Competing Interests section

Data precision has been standardized to 2 decimal places throughout the manuscript

Duplicate text “contribute cont to” has been corrected

All plain text plus/minus have been changed to the symbol plus/minus.

________________________________________

7. PLOS authors have the option to publish the peer review history of their article (what does this mean?). If published, this will include your full peer review and any attached files.

If you choose “no”, your identity will remain anonymous but your review may still be made public.

Attachment Submitted filename: Response_to_Reviewers_auresp_2.docx

# Peer review: Decision Letter 2

**DOI:** 10.1371/journal.pone.0361052.r005

**Authors:** Yalong Dang

8 Sep 2026

Dear Dr. Bursell,

Thank you for submitting your manuscript to PLOS One. After careful consideration, we feel that it has merit but does not fully meet PLOS One’s publication criteria as it currently stands. Therefore, we invite you to submit a revised version of the manuscript that addresses the points raised during the review process.

Please submit your revised manuscript by Oct 23 2026 11:59PM. If you will need more time than this to complete your revisions, please reply to this message or contact the journal office at plosone@plos.org. When you're ready to submit your revision, log on to https://www.editorialmanager.com/pone/ and select the 'Submissions Needing Revision' folder to locate your manuscript file.

- A letter that responds to each point raised by the academic editor and reviewer(s). You should upload this letter as a separate file labeled 'Response to Reviewers'.
- A marked-up copy of your manuscript that highlights changes made to the original version. You should upload this as a separate file labeled 'Revised Manuscript with Track Changes'.
- An unmarked version of your revised paper without tracked changes. You should upload this as a separate file labeled 'Manuscript'.

If you would like to make changes to your financial disclosure, please include your updated statement in your cover letter. Guidelines for resubmitting your figure files are available below the reviewer comments at the end of this letter.

If applicable, we recommend that you deposit your laboratory protocols in protocols.io to enhance the reproducibility of your results. Protocols.io assigns your protocol its own identifier (DOI) so that it can be cited independently in the future. For instructions see: https://journals.plos.org/plosone/s/submission-guidelines#loc-laboratory-protocols. Additionally, PLOS One offers an option for publishing peer-reviewed Lab Protocol articles, which describe protocols hosted on protocols.io. Read more information on sharing protocols at https://plos.org/protocols?utm_medium=editorial-email&utm_source=authorletters&utm_campaign=protocols.

As the corresponding author, your ORCID iD is verified in the submission system and will appear in the published article. PLOS supports the use of ORCID, and we encourage all coauthors to register for an ORCID iD and use it as well. Please encourage your coauthors to verify their ORCID iD within the submission system before final acceptance, as unverified ORCID iDs will not appear in the published article. *Only*  the individual author can complete the verification step; PLOS staff *cannot*  verify ORCID iDs on behalf of authors.

We look forward to receiving your revised manuscript.

Kind regards,

Yalong Dang

Academic Editor

PLOS One

Journal Requirements:

If the reviewer comments include a recommendation to cite specific previously published works, please review and evaluate these publications to determine whether they are relevant and should be cited. There is no requirement to cite these works unless the editor has indicated otherwise.

Please review your reference list to ensure that it is complete and correct. If you have cited papers that have been retracted, please include the rationale for doing so in the manuscript text, or remove these references and replace them with relevant current references. Any changes to the reference list should be mentioned in the rebuttal letter that accompanies your revised manuscript. If you need to cite a retracted article, indicate the article’s retracted status in the References list and also include a citation and full reference for the retraction notice.

[Note: HTML markup is below. Please do not edit.]

Reviewers' comments:

Reviewer's Responses to Questions

**Comments to the Author**

Reviewer #3: All comments have been addressed

**********

2. Is the manuscript technically sound, and do the data support the conclusions?

Reviewer #3: Yes

**********

3. Has the statistical analysis been performed appropriately and rigorously?

Reviewer #3: Yes

**********

4. Have the authors made all data underlying the findings in their manuscript fully available?

The PLOS Data policy

Reviewer #3: Yes

**********

5. Is the manuscript presented in an intelligible fashion and written in standard English?

Reviewer #3: Yes

**********

Reviewer #3: The scientific content is sound, the statistical analyses are appropriate, and the conclusions are well supported by the data. Only a few typographical and formatting details remain, which can be efficiently addressed during the proof stage. I congratulate the authors on a rigorous and valuable contribution to the field of diabetic eye care.

Discussion, Line 221 Typographical error: "foref" Change to "for"

Table 6, Footnote Extra dash at end: "male = 0 -" Revise to "male = 0" or "sex = female" for clarity

Abstract Data precision previously inconsistent Confirmed that all proportions now use two decimal places (e.g., 0.47) – acceptable

Abstract Data precision previously inconsistent Confirmed that all proportions now use two decimal places (e.g., 0.47) – acceptable

**********

what does this mean?). If published, this will include your full peer review and any attached files.

If you choose “no”, your identity will remain anonymous but your review may still be made public.

**Do you want your identity to be public for this peer review?**  For information about this choice, including consent withdrawal, please see our Privacy Policy

Reviewer #3: No

**********

[NOTE: If reviewer comments were submitted as an attachment file, they will be attached to this email and accessible via the submission site. Please log into your account, locate the manuscript record, and check for the action link "View Attachments". If this link does not appear, there are no attachment files.]

To ensure your figures meet our technical requirements, please review our figure guidelines: https://journals.plos.org/plosone/s/figures

You may also use PLOS’s free figure tool, NAAS, to help you prepare publication quality figures: https://journals.plos.org/plosone/s/figures#loc-tools-for-figure-preparation.

NAAS will assess whether your figures meet our technical requirements by comparing each figure against our figure specifications.

# Author comment: Author response to Decision Letter 3

**DOI:** 10.1371/journal.pone.0361052.r006

10 Sep 2026

Reviewer #3: The scientific content is sound, the statistical analyses are appropriate, and the conclusions are well supported by the data. Only a few typographical and formatting details remain, which can be efficiently addressed during the proof stage. I congratulate the authors on a rigorous and valuable contribution to the field of diabetic eye care.

Discussion, Line 221 Typographical error: "foref" Change to "for"

The typographical error was tracked in the track changes of the prior manuscript revision and reads as “for” in the cleaned manuscript. For clarity the revised manuscript tracks the change and still reads as “for” in the cleaned revised manuscript

Table 6, Footnote Extra dash at end: "male = 0 -" Revise to "male = 0" or "sex = female" for clarity

The footnote of Table 6 has been changed to clarify the gender designation code. It now reads “male = 0, female = 1”

Abstract Data precision previously inconsistent Confirmed that all proportions now use two decimal places (e.g., 0.47) – acceptable

Confirmed as acceptable

Abstract Data precision previously inconsistent Confirmed that all proportions now use two decimal places (e.g., 0.47) – acceptable

Confirmed as acceptable

________________________________________

Attachment Submitted filename: Response_to_Reviewers_auresp_3.docx

# Peer review: Decision Letter 3

**DOI:** 10.1371/journal.pone.0361052.r007

**Authors:** Yalong Dang

5 Oct 2026

Rates of Nonproliferative Diabetic Retinopathy (NPDR) regression among patients with Very Mild or Mild NPDR in an Indian Health Service teleophthalmology program, 2015 - 2020

PONE-D-26-08283R3

Dear Dr. Bursell,

We’re pleased to inform you that your manuscript has been judged scientifically suitable for publication and will be formally accepted for publication once it meets all outstanding technical requirements.

Within one week, you’ll receive an e-mail detailing the required amendments. When these have been addressed, you’ll receive a formal acceptance letter and your manuscript will be scheduled for publication.

An invoice will be generated when your article is formally accepted. Please note, if your institution has a publishing partnership with PLOS and your article meets the relevant criteria, all or part of your publication costs will be covered. Please make sure your user information is up-to-date by logging into Editorial Manager at Editorial Manager® and clicking the ‘Update My Information' link at the top of the page. For questions related to billing, please contact billing support.

If your institution or institutions have a press office, please notify them about your upcoming paper to help maximize its impact. If they’ll be preparing press materials, please inform our press team as soon as possible -- no later than 48 hours after receiving the formal acceptance. Your manuscript will remain under strict press embargo until 2 pm Eastern Time on the date of publication. For more information, please contact onepress@plos.org.

Kind regards,

Yalong Dang

Academic Editor

PLOS One

Additional Editor Comments (optional):

Reviewers' comments:

Reviewer's Responses to Questions

**Comments to the Author**

Reviewer #3: All comments have been addressed

**********

2. Is the manuscript technically sound, and do the data support the conclusions?

Reviewer #3: Yes

**********

3. Has the statistical analysis been performed appropriately and rigorously?

Reviewer #3: Yes

**********

4. Have the authors made all data underlying the findings in their manuscript fully available?

The PLOS Data policy

Reviewer #3: Yes

**********

5. Is the manuscript presented in an intelligible fashion and written in standard English?

Reviewer #3: Yes

**********

Reviewer #3: (No Response)

**********

what does this mean?). If published, this will include your full peer review and any attached files.

If you choose “no”, your identity will remain anonymous but your review may still be made public.

**Do you want your identity to be public for this peer review?**  For information about this choice, including consent withdrawal, please see our Privacy Policy

Reviewer #3: No

**********

# Editor report: Acceptance letter

**DOI:** 10.1371/journal.pone.0361052.r008

**Authors:** Yalong Dang

PONE-D-26-08283R3

PLOS One

Dear Dr. Bursell,

I'm pleased to inform you that your manuscript has been deemed suitable for publication in PLOS One. Congratulations! Your manuscript is now being handed over to our production team.

At this stage, our production department will prepare your paper for publication. This includes ensuring the following:

* All references, tables, and figures are properly cited

* All relevant supporting information is included in the manuscript submission,

* There are no issues that prevent the paper from being properly typeset

You will receive further instructions from the production team, including instructions on how to review your proof when it is ready. Please keep in mind that we are working through a large volume of accepted articles, so please give us a few days to review your paper and let you know the next and final steps.

Lastly, if your institution or institutions have a press office, please let them know about your upcoming paper now to help maximize its impact. If they'll be preparing press materials, please inform our press team within the next 48 hours. Your manuscript will remain under strict press embargo until 2 pm Eastern Time on the date of publication. For more information, please contact onepress@plos.org.

You will receive an invoice from PLOS for your publication fee after your manuscript has reached the completed accept phase. If you receive an email requesting payment before acceptance or for any other service, this may be a phishing scheme. Learn how to identify phishing emails and protect your accounts at https://explore.plos.org/phishing.

If we can help with anything else, please email us at customercare@plos.org.

Thank you for submitting your work to PLOS One and supporting open access.

Kind regards,

PLOS One Editorial Office Staff

on behalf of

Dr Yalong Dang

Academic Editor

PLOS One
